# Germacrone Derivatives as new Insecticidal and Acaricidal Compounds: A Structure-Activity Relationship

**DOI:** 10.3390/molecules24162898

**Published:** 2019-08-09

**Authors:** Alberto Galisteo Pretel, Helena Pérez del Pulgar, Estela Guerrero de León, José Luis López-Pérez, A. Sonia Olmeda, Azucena Gonzalez-Coloma, Alejandro F. Barrero, José Francisco Quílez del Moral

**Affiliations:** 1 Department of Organic Chemistry, Institute of Biotechnology, University of Granada, 18071 Granada, Spain; 2 Department of Pharmacology, Faculty of Medicine, University of Panama, Panama 07156, Panama; 3 Department of Pharmaceutical Sciences, IBSAL-CIETUS, University of Salamanca, 37007 Salamanca, Spain; 4 Faculty of Veterinary, Complutense University of Madrid (UCM), 28040 Madrid, Spain; 5 Institute of Agricultural Sciences, CSIC, 28006 Madrid, Spain

**Keywords:** natural products, organic synthesis, bioactive compounds, insecticidal, ixodicidal

## Abstract

Currently, the use of synthetic pesticides is the main method of plant protection applied in agri- and horticulture. However, its excessive use leads to the development of pesticide resistance, a contamination of the environment, toxicity to non-target organisms, and risks for human health. With the ultimate aim of contributing to the develop of a more sustainable pest management, we used the natural product germacrone (**compound** **1**), reported to possess significant insecticidal activity, as starting material for the generation of molecular diversity (**2**–**24**). Some of the generated derivatives are natural compounds, such as 1,10-epoxygermacrone (**2**), 4,5-epoxygermacrone (**3**), gajutsulactone A (**7**), germacrol (**11**), isogermacrone (**14**), 9-hydroxyeudesma-3,7(11)dien-6-one (**19**), eudesma-4,7(11),dien-8-one (**20**), eudesma-3,7(11)-dien-8-one (**21**) and eudesma-4(15),7(11)-dien-8-one (**22**). Compounds, 7,11-9,10-diepoxigermacr-4,5-en-8-ol (**17)**, 7,11-epoxieudesma-4,7(11)-dien-8-one (**23**) and 7,11-epoxieudesma-3,7(11)-dien-8-one (**24**) are described for the first time. The biocidal activity of most of these compounds was assayed against the tick *Hyalomma lusitanicum.* The acaricidal effects of compound **24** were four times higher than that of germacrone (**1**). Compound **2** is an insect antifeedant a thousand times more potent than germacrone against *Rhopalosiphum padi*, which makes this substance a promising selective antifeedant against this cereal pest.

## 1. Introduction

Germacrone (**1**) is a sesquiterpene isolated in significant amounts from the essential oil of *Geranium macrorhyzum* (Hungary chemotype) [1]. This essential oil shows antifeedant effects against aphids and ixodicidal activity against the tick *Hyalomma lusitanicum* along with antifeedant effects against the insect pests *Spodoptera littoralis* Boisd, *Myzus persicae* Sulz, and *Rhopalosiphum padi*, with compound **1** being, to a large extent, responsible for these activities [1]. Additionally, Benelli et al. report both the insecticidal activity of germacrone (**1**) against mosquito larvae (*Culex quinquefasciatus*) [2], and also its acaricidal effects against *Tetranychus urticae* [3].

Regarding the contact toxicity of germacrone against *Tribolium castaneum*, García et al. report that germacrone results in not being toxic [4], whereas Liang et al. confirm its contact toxicity [5]. The difference in the used concentrations may be argued to rationalize this discrepancy.

Germacrol (**2**), the alcohol resulting from the reduction of the carbonyl group of germacrone, results active in choice bioassays against *T. castaneum*, presenting more activity that germacrone (**1**) [4]. Germacrone (**1**) is also used as starting material for the synthesis of 9-methylgermacrene to be used in mosquito control compositions [6].

The significance of the activities reported for germacrone (**1**) prompted Navarro-Rocha et al. to address the domestication and cultivation of its natural source, *G. macrorhyzum* (Hungary chemotype), for the sustainable production of this natural product. In this context, and after considering the particular functionality of this molecule [7,8], we selected germacrone as a series lead for the development of both new ixodicidal agents and new aphid antifeedants. Thus, 23 compounds were generated from germacrone (**1**), mostly after one or two chemical steps, and their biocide activity against the target species was tested and compared to that of germacrone (**1**).

## 2. Results and Discussion

**Figure 1:**
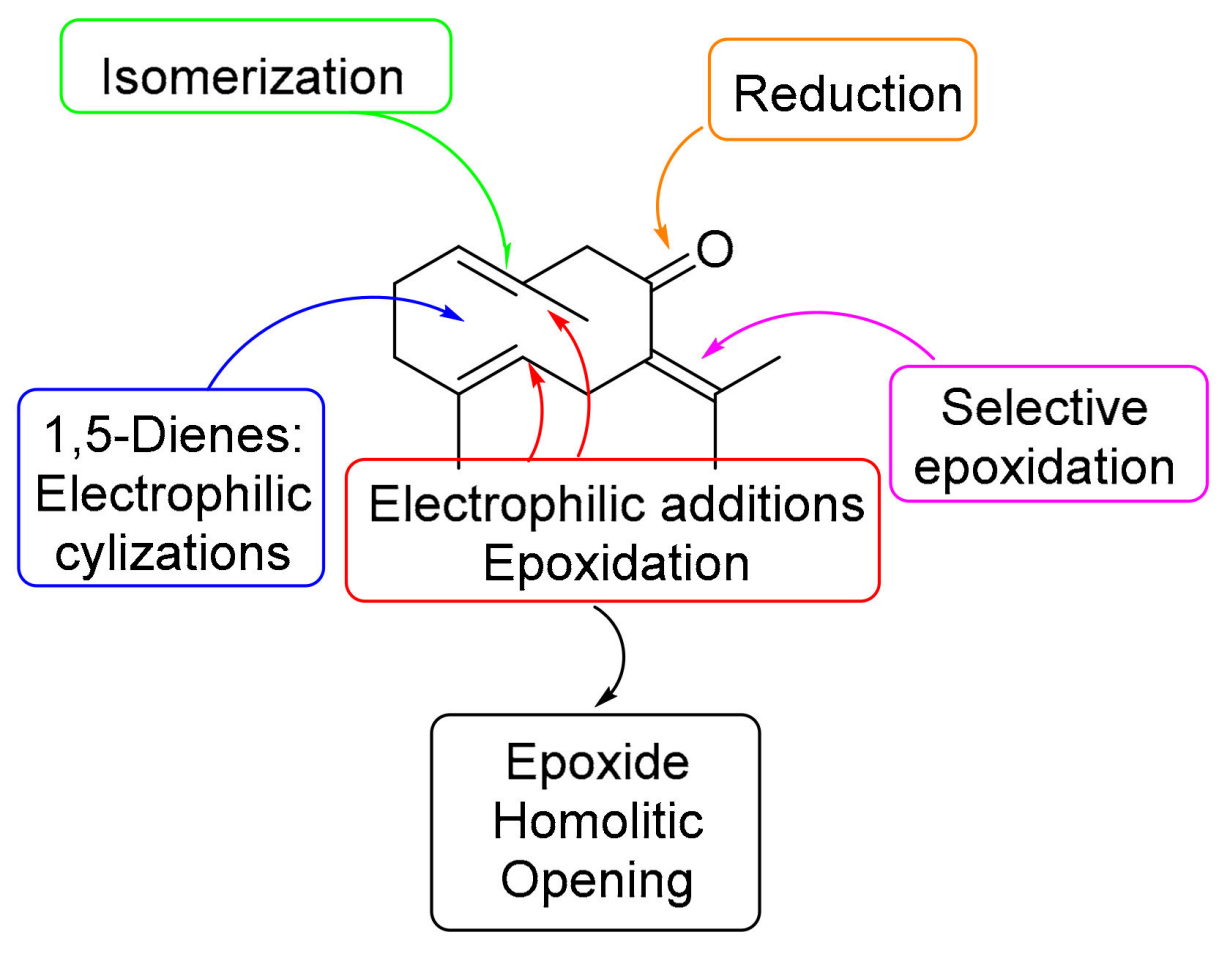
Generation of structural diversity from germacrone (**1**).

Germacrone (**1**) presents an appropriate molecular structure and functionality to be used in a reagent-based approach as a source of chemical diversity. Figure 1 shows some of the transformations planned to be carried out.

Some of the compounds tested were synthesized following protocols previously reported by some of us, and include only one or two synthetic steps from germacrone (**1**) [7].

**Scheme 1:**
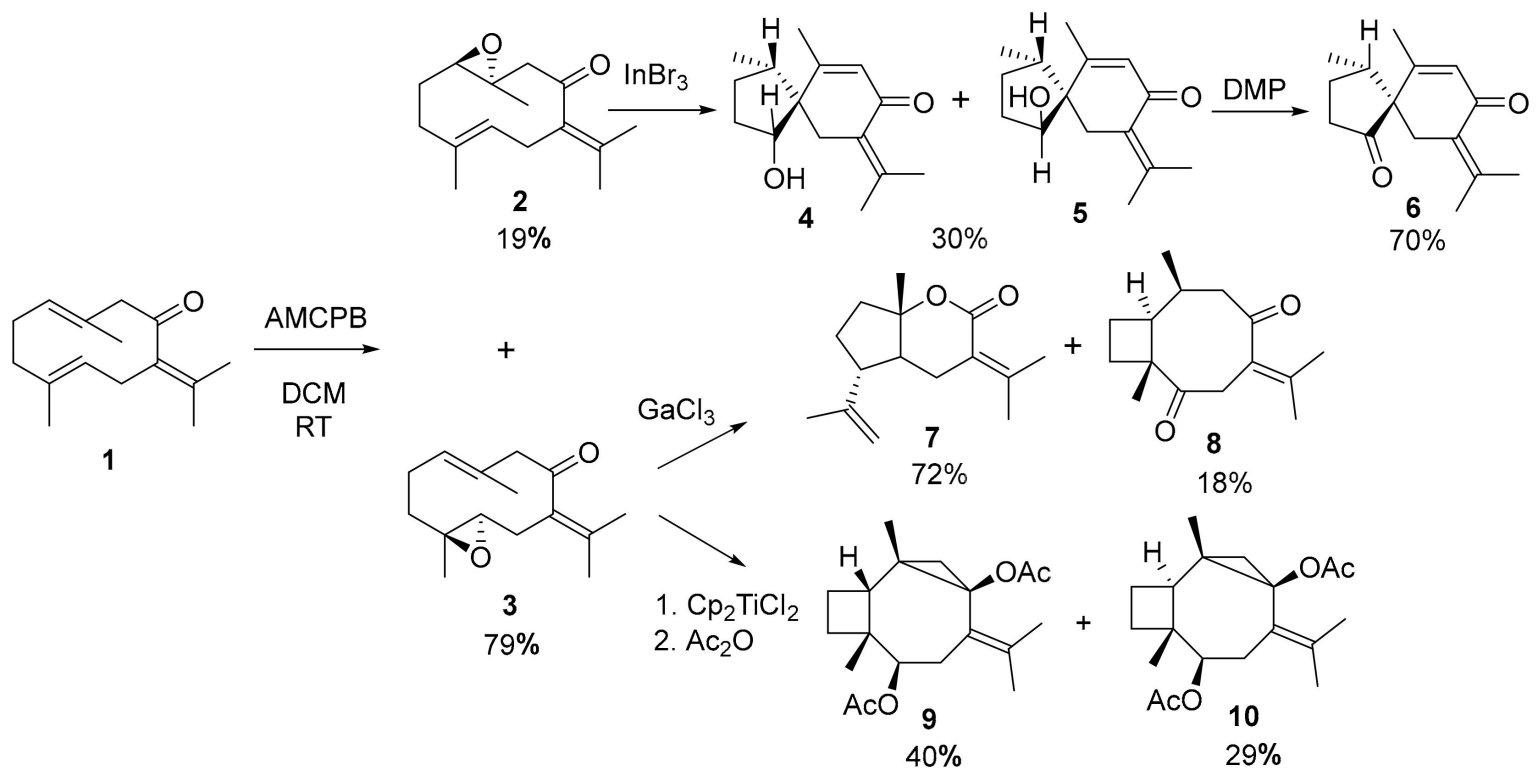
Synthesis of racemic **2**–**10** from germacrone (**1**). Yield of the isolated products is shown.

The synthesis of compounds **2**–**10** are summarized in Scheme 1. Direct epoxidation of germacrone (**1**) with mCPBA yields a mixture of epoxides **2**–**3** in a 4:1 ratio. Epoxides **2** and **3** are also natural products obtained from the rhizomes of several Curcuma species [9,10,11]. The ionic opening of 1(10)-epoxygermacrone (**2**) triggered by the Lewis acid InBr_3_ results in the generation of the spiro-alcohols **4**–**5** (30%). Dess-Martin oxidation of **4**–**5** produces the corresponding ketone **6** with an acceptable yield. Treatment of 4,5-epoxygermacrone (**3**) with GaCl_3_ as a Lewis acid causes the opening of **2** to give a natural product known as gajutsulactone A (**7**) (72%) [12], together with minor amounts of diketone (**8**) (18%). On the other hand, the treatment of **3** with titanocene monochloride and the subsequent acetylation produces the mixture of acetates **9**–**10**.

**Scheme 2:**
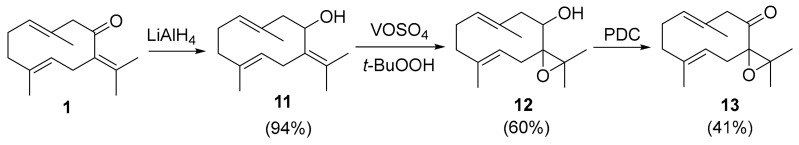
Synthesis of racemic epoxide **13**. Yield of the isolated products is shown.

Attempts to achieve the chemoselective epoxidation of the exocyclic double bond of **1** using H_2_O_2_/NaOH in different solvents were unsuccessful, since the isomerization of the double bond 1,(**10**) took place before the start of the epoxidation process. Compound **13** was then obtained following the protocol described by Enev and Tsankova [13], although varying the vanadium species used for the C7–C11 selective epoxidation of **11** (Scheme 2).

**Figure 2:**
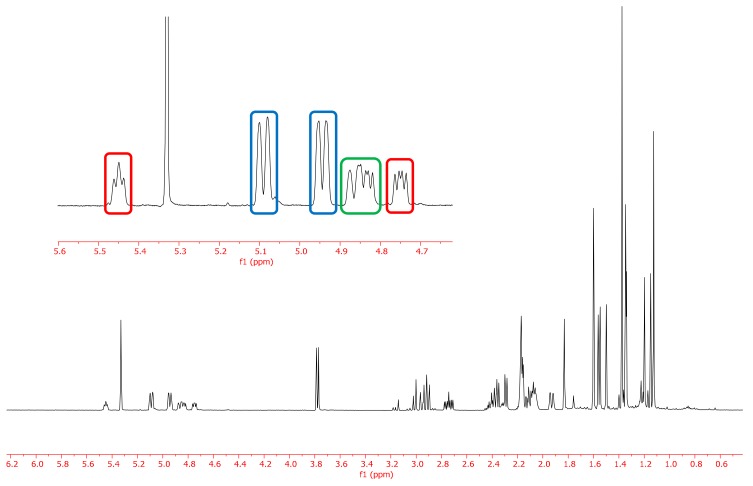
^1^H-NMR spectrum of epoxide **13** at −20 °C. Grouped in red, blue and green are the three sets of olefinic signals attributable to the three conformers of this molecule existing in solution.

Epoxide **13** shows in its ^1^H-NMR at room temperature three sets of signals that can be attributed to three energetically similar conformers, with relatively high barriers of interconnection between them. The three sets of signals appear better resolved when the NMR spectrum was performed at −20 °C (Figure 2, see also Supplementary Material). The ability of the ten-membered ring of the germacradiene species to undergo conformational changes is well known, and has been the subject of multiple computational studies [14,15,16,17,18,19]. This conformational flexibility plays an important role in their biosynthetic transformation to other sesquiterpene skeletons [20,21], and in the reactivity of these compounds, since the spatial disposition of the different conformers determines the outcome of the corresponding reactions [22,23].

**Figure 3:**
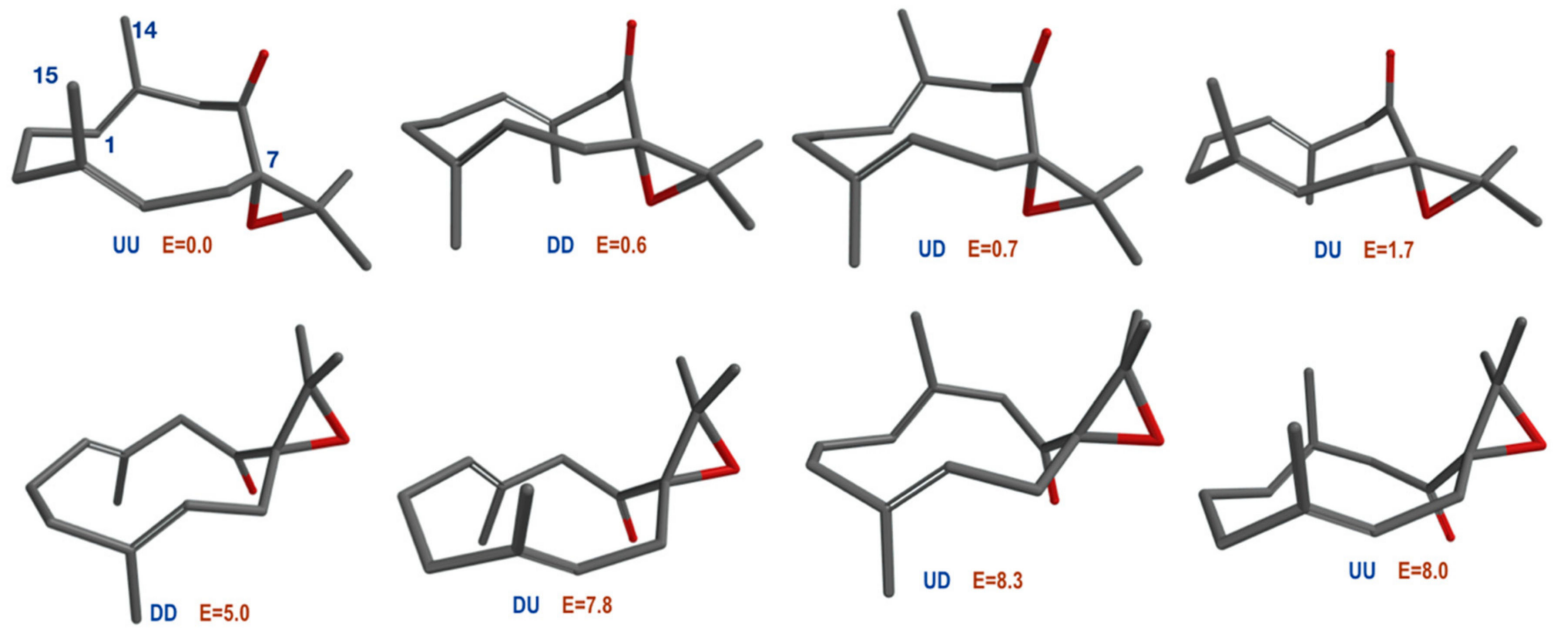
Main conformers of the 7*S*-epimer of epoxide **13**. Relative free energies (red) to the conformer UU in kcal/mol (B3LYP-6-31+G(d,p)).

This prompted us to undertake a computational study of compound **13**. Initially, a conformational hybrid search method recommended for macrocyclic systems [24] and implemented in Macromodel [25], allowed the detection of 8 conformers for each of the C7 epimers of compound **13**, which were subsequently optimized with Gaussian16 [26] using the B3LYP/6-31+G(d,p) [27,28] level of theory. The 8 main conformers found for the 7*S*-epimer are represented in Figure 3. These conformers are described as up–up (UU), up–down (UD), down–up (DU) and down–down (DD), depending upon the orientation of the C14 and C15 methyl groups, in accordance with the terminology introduced by Watson and Kashyap [19].

The four conformations with the down disposition of ketone at C8 are in-between 5 to 8 kcal/mol less stable than the global minimum conformer UU with the ketone at C8 also up, so their population contribution in the conformational equilibrium of this compound will be practically null, according to the Boltzman distribution equation. From the four lower energy conforms, DD and UD conformers possess free energy values 0.7–0.8 kcal/mol higher than the global minimum UU, while the fourth conformer is 1.7 kcal/mol higher in its free energy value. These data would point to the possibility of the coexistence of different conformations in its structure at room temperature.

**Figure 4:**
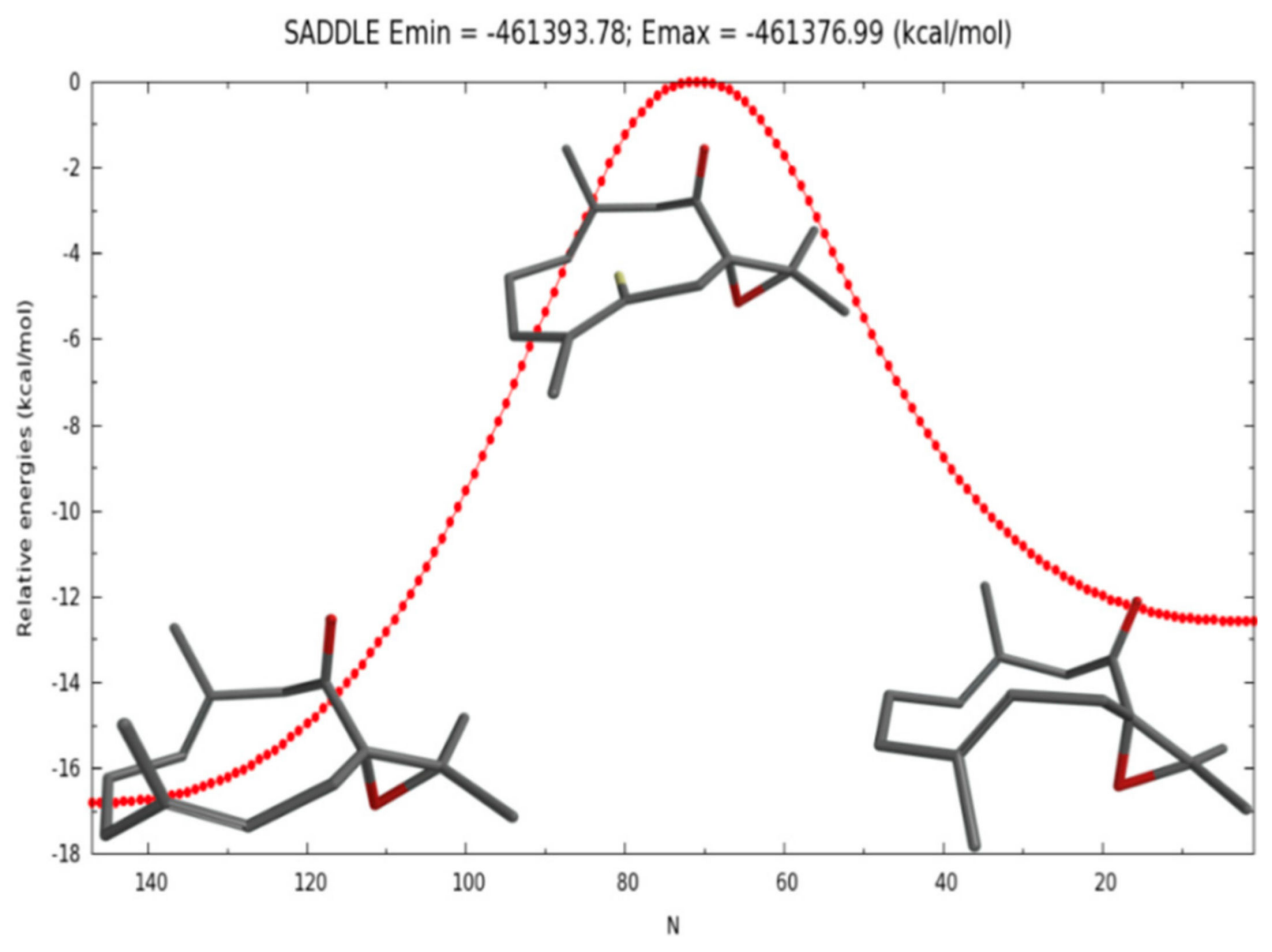
Barrier of exchange between conformations UU and UD and the transition state between both of them.

**Figure 5:**
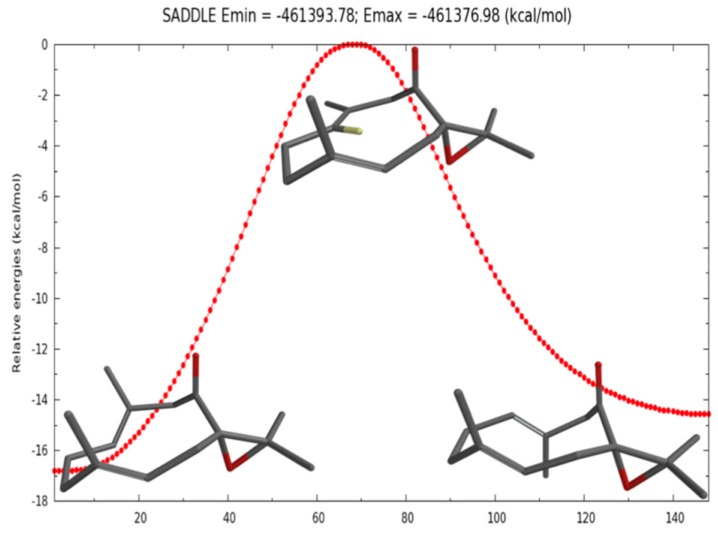
Barrier of exchange between conformations UU and DU and the transition state between both of them.

**Figure 6:**
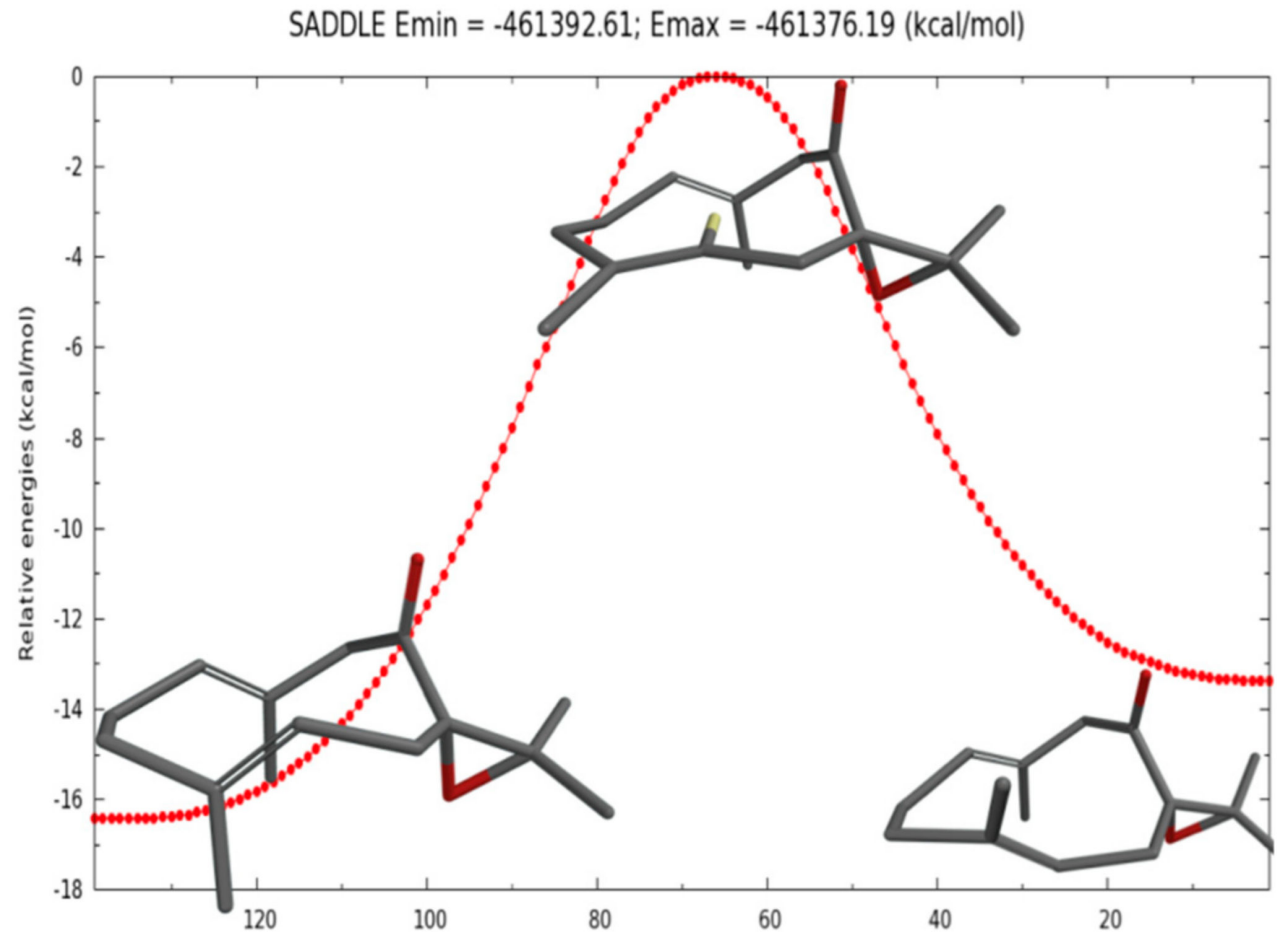
Barrier of exchange between conformations DD and DU and the transition state between both of them.

In order to justify the observation of three sets of signals in the ^1^H-NMR spectrum of epoxide **13**, conformational exchange studies were undertaken between the three lowest-energy conformations by rotation around the two planes of both double bonds present in the molecule. These barriers of exchange between the three conformations (Figure 4, Figure 5 and Figure 6), (although surmountable at room temperature), are relatively high (around 16 kcal/mol), which would suggest a low rate of exchange between conformers, and therefore a differentiation in the NMR spectrum.

Finally, the theoretical ^1^H–^1^H nuclear coupling constants (J_H_–_H_) were calculated by applying the scaled proton-proton Bally and Rablen methodology [29] for the three most stable conformers of compound **13**. These values were compared with the experimental data measured in dichloromethane at −10 °C. Gratifyingly, a remarkable coincidence was observed between the theoretical and experimental data (Table 1).

**Figure 7:**
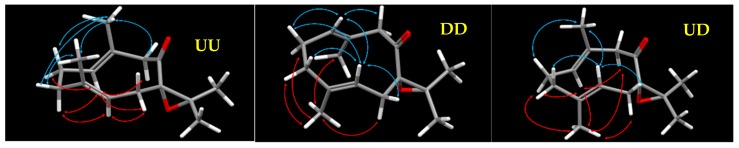
Observed NOEs (indicated by blue and red arrows) for the three conformers of **13** (UU, DD and UD) existing in solution.

A final support to both experimental and theoretical data was found after analyzing the NOEDIFF experiments performed in compound **13** (Figure 7). These data also suggests that the DD, UU and UD conformers were the three ones existing in dichloromethane solution for this substance.

**Scheme 3:**
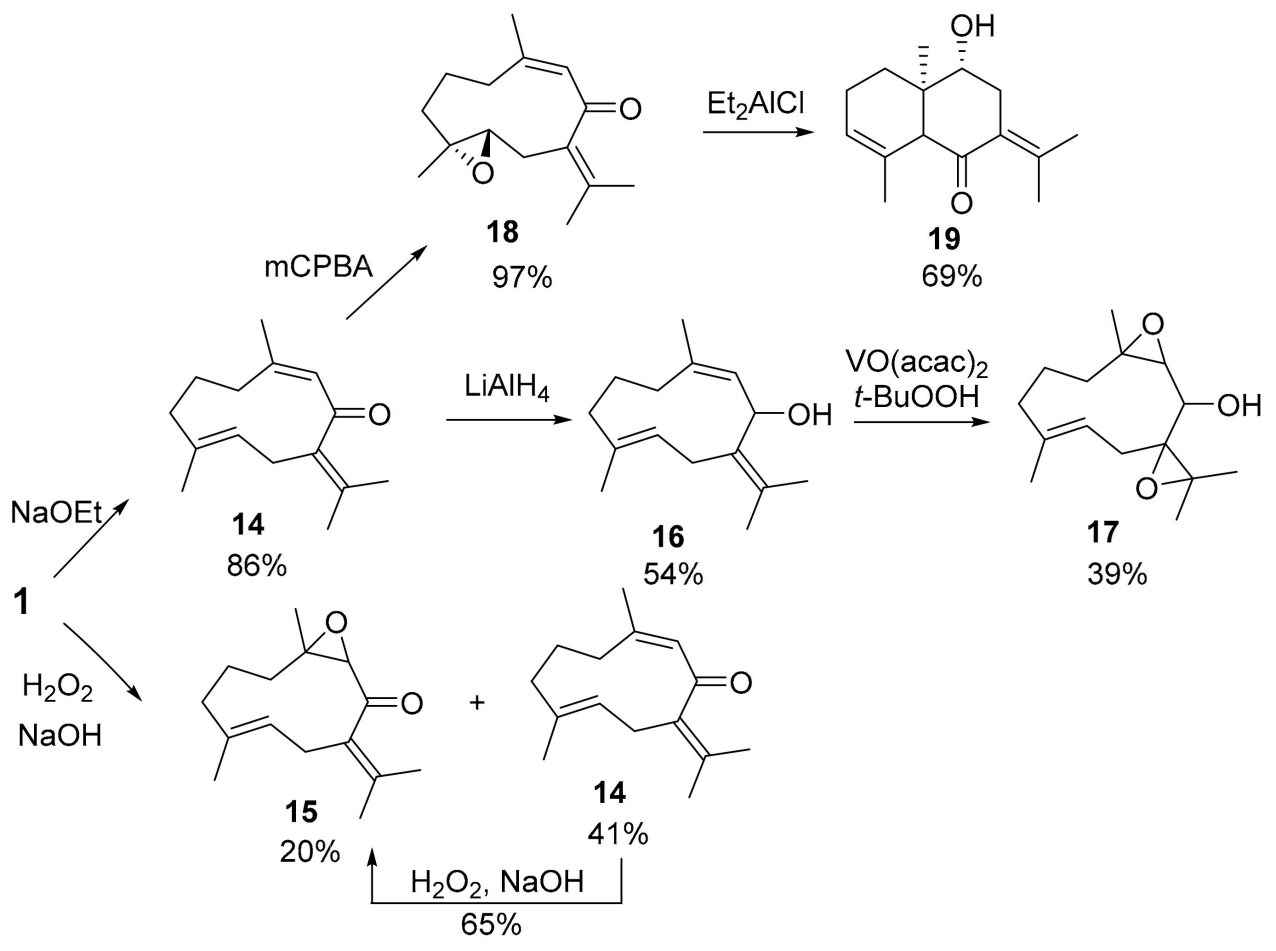
Synthesis of racemic isogermacrone derivatives. The yield of isolated products is shown.

Getting back to the preparation of derivatives of **1**, isogermacrone (**14**) was obtained from **1** after NaOEt-mediated isomerization to afford a 86% yield of the natural product **14** [30]. The chemoselective epoxidation of the conjugated double bond of **14** employing alkaline H_2_O_2_ in EtOH affords epoxide **15** (20%) (Scheme 3). Epoxide **15** could be generated more efficiently by direct epoxidation of isogermacrone with H_2_O_2_ in EtOH (65%).

LAH-mediated reduction of isogermacrone (**14**) yields isogermacrol (**16**) (54%). The NMR spectra of this molecule only shows acceptable resolution when performed at high temperature. The lack of resolution at room temperature can be attributed to the existence of coalescence phenomena at this temperature. The epoxydation of isogermacrol (**16**) was not selective and diepoxide **17** was originated after a vanadium-catalyzed double epoxidation. The treatment of isogermacrone (**14**) with mCPBA leads selectively to 4,5-epoxyderivative **18**, which was converted to eudesmanone **19**, via a cyclization process triggered by diethylaluminium chloride [10]. Compound **19** is a natural product isolated from Curcuma phaeocaulis [31].

**Scheme 4:**
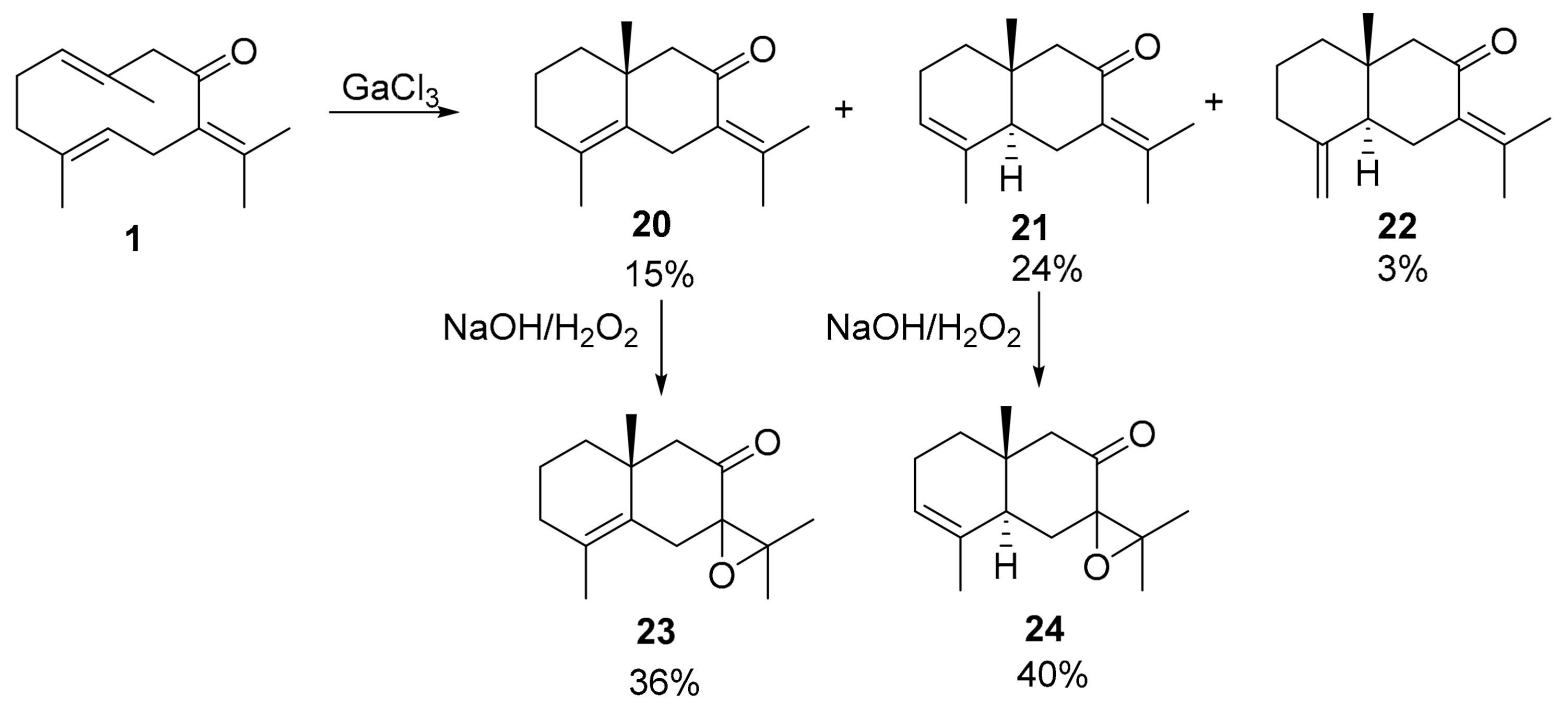
Synthesis of racemic epoxyeudesmenes **23** and **24**. Yield of isolated products is shown.

Finally, epoxyeudesmenes **23** and **24** are obtained again in only two steps. The first one involves a Lewis acid-mediated cyclization of germacrone (**1**) to afford eusdesmadienes **20** (15%) and **21** (24%) and **22** (3%). These three eudesmanes are natural products isolated from different essential oils [32,33,34]. The epoxydation of **20** and **21** with the alkaline peroxide system H_2_O_2_/NaOH affords epoxides **23** and **24** with acceptable yields (Scheme 4).

At this point, we tested the acaricidal of germacrone (**1**) and of most of its derivatives against the hard tick *H. lusitanicum*. The results obtained are shown in Table 2.

The most active ixodicidal compounds result in being epoxyeudesmene **24** and isogermacrone **14**, and then, germacrone (**1**), 7,11-epoxigermacrone (**13**), and 4,5-epoxigermacrone (**3**). The bicyclic diketone **8**, 1,10-epoxigermacrona (**2**), 9,10-epoxiisogermacrona (**15**), lactone **7** (gajutsulactone A) and eudesmane **19** were the less active derivatives. Epoxyeudesmene **24**, presenting an LD_50_ four times higher than that of germacrone (**1**), may be considered a promising ixodicidal candidate.

From a structure-activity approach, isogermacrone (**14**) is closely-related to germacrone (**1**), whereas compounds **13** and **24** contain an epoxide close the carbonyl group, instead of a double bond. All this seems to indicate that the presence of an epoxy function located in the α,β position to the carbonyl group enhances the ixodicidal activity. Both substances may be considered analogs of the sesquiterpene with eremophilane skeleton ligudicine reported as a strong ixodicidal [35]. On the other hand, the presence of an epoxide at C4–C5 (**3**) does not affect the activity of these compounds, nor does the existence of a keto group in a cyclooctane ring (**8**). In these cases, the enone functionality must be responsible for the observed activity. Finally, other structural modifications in **1**, such as the presence of a lactone instead of the ketone, or the isomerization of the double bond, cause a significant decrease of the activity. Nine molecules (**4**–**6**, **9**–**10** and **17**–**21**) result in being inactive. In this case, the disappearance of the enone (**9**–**10**) or the presence of the highly strained spirane system may well be the causes of the inactivity. The antifeedant effects of these germacrone derivatives against the insect pest *S. littoralis* Boisd, *M. persicae* Sulz, and *R. padi* L were also tested (Table 3). None of the compounds assayed improve the moderate-high activity of germacrone against *S. littoralis*. Only diketone **8** presents a similar activity, whereas the epoxyderivatives **2** and **3**, the diepoxide **17** and the acetylated tricycles **9** and **10** result in being less active than germacrone. Similar results were found with *M. persicae*.

Thus, only **15** shows activity comparable to that of germacrone. Four compounds, **2**, **8**, **10** and **13**, present low-moderate activity, and the remaining ones were inactive, or show very low activity.

Much more interesting are the results obtained against *R. padi*. Thus, two compounds, 1,10-epoxygermacrone (**2**) and gajutsulactone A (**7**), result in being very active against this aphid (EC_50_ values: 6 × 10^−3^ and 2 × 10^−2^). These data convert these two substances into promising selective antifeedants against this cereal pest.

Four other molecules (**3**, **8**, **17** and **23**) show high activity. Finally, compounds **13** and **15** present moderate activity, while the remaining compounds show no relevant effect.

## 3. Materials and Methods

### 3.1. Instruments and Chemicals

Silica gel SDS 60 (35–70 μm) was used for flash column chromatography. NMR spectra were acquired with Varian Direct-Drive 600 (^1^H 600 MHz/^13^C 150 MHz), Varian Direct-Drive 500 (^1^H 500 MHz/^13^C 125 MHz) and Varian Direct-Drive 400 (^1^H 400 MHz/^13^C 100 MHz). Accurate mass determinations were achieved with a SYNAPT G2-Si Q-TOF mass spectrometer (Waters, Milford, MA, USA) equipped with high-efficiency T-Wave ion mobility and an orthogonal Z–spray™ electrospray ionization (ESI) source. MassLynx v.4.1 software was used for HRMS instrument control, peak detection, and integration. The reactions were monitored by TLC, which were performed on 0.25-mm E. Merck silica gel plates (60F-254) and involves the use of UV light for visualization and solutions of phosphomolybdic acid in EtOH, and heat as the developing agents. HPLC with UV light and RI detection was also used. The reagents were purchased at the highest quality that was commercially available, and were used without further purification.

### 3.2. Synthesis

Compounds **1**–**6**, **9**–**10**, **14**, and **18**–**19** were prepared as described previously by some of us [7].

#### 3.2.1. Cyclization of 4,5-epoxygermacrone (**3**) with GaCl_3_

To a solution of 4,5-epoxygermacrone (**3**) (150mg, 0.64 mmol) in dry DCM (10 mL) GaCl_3_ (11 mg, 0.1 mmol) was added under an argon atmosphere at −20 °C. This mixture was stirred for 90 min and monitored by thin-layer chromatography analysis. Upon consumption of the starting material the mixture was evaporated in vacuo. The crude products were purified by flash chromatography (H:MTBE, 7:3) to obtain 108 mg of **7** (72%) and 27 mg of **8** (18%). Compounds **7** and **8** were described previously [7].

#### 3.2.2. Synthesis of 7,11-epoxygermacrone (**13**)

To a solution of **1** (1.5 g, 6.8 mmol) in dry THF (20 mL) under argon atmosphere was added 200 mg of LiAlH_4_ at −10 °C. The reaction mixture was stirred at this temperature for 10 min and monitored by thin-layer chromatography analysis. Upon consumption of the starting material, 0.15 mL of water, a solution of 6 N NaOH (0.15 mL) and 0.6 mL of water were added dropwise. The mixture was filtered over silica gel and Na_2_SO_4_ (2:1) and washed with MTBE. The reaction was evaporated in vacuo to obtain 1.4 g of **11** (94%). Spectroscopic data of germacrol (**11**) were previously described [4].

To a solution of germacrol (**11**) (1000 mg, 4.54 mmol) in benzene (25 mL) under an argon atmosphere was added 37 mg of VOSO_4_ (0.23 mmol). After the reaction mixture was heated at reflux temperature for 10 min, 1.6 mL of t-BuOOH (9.08 mmol) was added. The reaction was then refluxed for 30 additional min and monitored by thin-layer chromatography. Upon consumption of the starting material, the mixture was diluted with 250 mL of EtOAc, washed with a saturated solution of NaHCO_3_ (3 × 100 mL) and brine (3 × 100 mL). The organic layer was dried over Na_2_SO_4_ and evaporated in vacuo. The crude product was purified by flash chromatography (H:MTBE, 9:1) to obtain 616 mg of 7,11-epoxyisogermacrol (**12**) (60%). ^1^H-NMR (400 MHz, CDCl_3_) δ 4.82 (dd, *J* = 11.8, 3.1 Hz, 1H), 4.51 (d, *J* = 11.3 Hz, 1H), 4.00 (d, *J* = 10.0 Hz, 1H), 2.63 (dd, *J* = 14.8, 11.3 Hz, 1H), 2.52 (d, *J* = 10.1 Hz, 1H), 2.44 (d, *J* = 13.0 Hz, 1H), 2.34 (td, *J* = 12.3, 4.9 Hz, 1H), 2.29–2.19 (m, 2H), 2.09 (dd, *J* = 12.4, 5.0 Hz, 1H), 2.00 (td, *J* = 12.1, 5.4 Hz, 1H), 1.60 (s, 3H), 1.55 (d, 6H), 1.46 (s, 3H). ^13^C-NMR (100 MHz, CDCl_3_) δ 134.52, 133.32, 129.78, 123.98, 72.48, 70.46, 63.38, 46.28, 38.73, 35.25, 25.22, 22.25, 21.20, 16.66, 16.39. HRMS (ESI+) *m*/*z* calculated for C_15_H_24_O_2_ [M + H]^+^ 237.1855, found 237.1838.

To a solution of **12** (160 mg, 0.68 mmol) in dry DMF (3.5 mL) under an argon atmosphere, 1.8 g of PDC (4.78 mmol) were added at 0 °C. The reaction mixture was stirred at this temperature for 30 min and monitored by thin-layer chromatography. Upon consumption of the starting material, the reaction was diluted in 35 mL of MTBE and washed with 35 mL of water. The aqueous layer was then washed with MTBE (3 × 20 mL). The combined organic layer was washed with brine (3 × 20 mL), dried over Na_2_SO_4_ and evaporated in vacuo. The crude product was purified by flash chromatography (H:MTBE, 6:1) to obtain 101 mg of 7,11-epoxygermacrone (**13**) (41%). ^1^H-NMR (500 MHz, CD_2_Cl_2_, −10 °C). Signals assigned to conformer UU: δ 5.09 (d, *J* = 12.3 Hz, 1H), 4.95 (d, *J* = 11.9 Hz, 1H), 3.8 (d, *J* = 9,6 Hz, 1H), 2.92 (t, *J* = 12.8 Hz, 1H), 2,38 (m, 1H), 2,29 (d, 9.5 Hz, 1H), 2.16 (m, 1H), 2.07 (m, 1H), 1.93 (dd, *J* = 13.9, 2.7 Hz, 1H), 1.6 (s, 3H), 1.35 (s, 3H). Signals assigned to conformer DD: δ 4.87 (*J* = 12.5 Hz, 1H), 4.83 (dd, *J* = 10.7, 7.0 Hz, 1H), 3.02–1.92 (m, 2H), 2.76 (dd, *J* = 14.3, 6.1 Hz, 1H), 2.41 (m, 1H), 2.17 (m,1H), 2.12 (m, 1H), 2.07 (m, 1H), 1.82 (s, 3H), 1.56 (s, 3H). Signals assigned to conformer UD: δ 5.45 (t, J = 7.5 Hz,1H) 4.74 (dd, *J* = 10.4,5.6 Hz,1H), 3.78 (d, *J* = 9.6 Hz, 1H), 2.72 (dd, *J* = 13.5, 5.9 Hz, 1H), 2.35 (d, *J* = 9,3 Hz, 1H), 2.34 (m,1H), 2.18 (m, 2H), 2.10 (m, 1H), 1.55 (s, 3H), 1.5 (s, 3H). ^13^C-NMR (125 MHz, CD_2_Cl_2_). Signals assigned to conformer UU: δ 207.5, 133.3, 122.4, 48.2, 37.8, 27.6, 23.5, 16.0, 15.3. Signals assigned to conformer DD: δ 207.0, 132.9, 122.9, 50.2, 28.5, 24.5, 23.3, 19.5, 15,7. Signals assigned to conformer UD: δ 206.9, 130.7, 121.4, 49.9, 28.7, 23.4, 15.8, 15.6. HRMS (ESI+) *m*/*z* calculated for C_15_H_22_O_2_ [M + H]^+^ 235.1698, found 235.1685.

#### 3.2.3. Synthesis of 9,10-epoxyisogermacrone (**15**)

To a solution of **1** (200 mg, 0.92 mmol) in EtOH (5 mL) was added 0.5 mL of H_2_O_2_ (30%) and 0.5 mL of a solution of 5N NaOH dropwise. The reaction mixture was stirred for 40 days at room temperature and monitored by thin-layer chromatography analysis. Upon consumption of the starting material, the mixture was diluted with 75 mL of MTBE, washed with brine (3 × 20 mL), dried over Na_2_SO_4_ and evaporated in vacuo. The crude product was purified by flash chromatography (H:MTBE, 9:1) to obtain 90 mg of **14** (41%) and 64 mg of **15** (20%). ^1^H-NMR (500 MHz, DMSO-*d*_6_, 85 °C) δ 5.17 (dd, *J* = 9.1, 6.5, 1H), 3.59 (s, 1H), 2.98 (dd, *J* = 15.1, 6.5 Hz, 1H), 2.90–2.85 (m, 1H), 2.16–2.11 (ddd, *J* = 13.6, 8.6, 6.1 Hz, 1H), 1.98 (dt, *J* = 12.9, 6.0 Hz, 1H), 1.74 (s, 3H), 1.71–1.70 (m, 1H), 1.68 (bs, 3H), 1.68–1.66 (m, 1H), 1.47 (s, 3H), 1.38 (s, 3H), 1.24–1.20 (m, 1H). ^13^C-NMR (125 MHz, DMSO-*d*_6_, 85 °C) δ 204.98, 136.74, 135.68, 130.70, 123.17, 67.99, 65.33, 37.59, 28.30, 27.44, 22.11, 21.72, 21.52, 19.97, 15.24. HRMS (ESI+) *m*/*z* calculated to C_15_H_23_O_2_ [M + H]^+^ 235.1698, found 235.1696.

To a solution of **14** (100 mg, 0.46 mmol) in 2.5 mL of EtOH) was added 0.25 mL of H_2_O_2_ (30%) and 0.25 mL of a solution of 5N NaOH dropwise. The reaction mixture was stirred for 48 h at room temperature, and then diluted with 40 mL of MTBE, washed with brine (3 × 10 mL), dried over Na_2_SO_4_ and evaporated in vacuo. The crude product was purified by flash chromatography (H:MTBE, 4:1) to obtain 70 mg of **15** (65%).

#### 3.2.4. Synthesis of 7,11-9,10-diepoxygermacr-4,5-en-8-ol (**17**)

To a solution of **14** (419 mg, 1.9 mmol) in dry THF (23 mL) under argon atmosphere, was added 108 mg of LiAlH_4_ at –10 °C. The reaction mixture was stirred at this temperature for 100 min and monitored by thin-layer chromatography. Upon consumption of the starting material the reaction mixture was diluted with MTBE, and then, 1 mL of water, 1 mL of 6N NaOH and 3 mL of water were added dropwise. Then the reaction was filtered over silica gel and Na_2_SO_4_ (2:1) and washed with EtOAc. The solvent was evaporated in vacuo. The crude product was purified by flash chromatography (H:MTBE, 4:1) to obtain 227 mg of isogermacrol (**16**) (54%). ^1^H-NMR (600 MHz, DMSO-*d*_6_, 80 °C) δ 5.26 (d, *J* = 9.7 Hz, 1H), 4.97 (bs, 1H), 4.75 (d, *J* = 9.6 Hz, 1H), 2.94 (dd, *J* = 14.5, 5.2 Hz, 1H), 2.65 (t, *J* = 12.3 Hz, 1H), 2.37 (ddd, *J* = 14.1, 9.9, 4.4 Hz, 1H), 2.07–1.98 (dp, *J* = 26.4, 6.5 Hz, 2H), 1.87 (d, *J* = 2.1 Hz, 3H), 1.83–1.77 (m, 1H), 1.64 (s, 3H), 1.62 (s, 3H), 1.60–1.57 (m, 2H), 1.50 (s, 3H). ^13^C-NMR (125 MHz, DMSO-*d*_6_, 80 °C): δ 132.4, 128.5, 72.6, 43.5, 41.3, 34.4, 31.5, 25.6, 24.6, 24.5, 22.5, 18.0. HRMS (ESI+) *m*/*z* calculated to C_15_H_25_O [M + H]^+^ 221.1905, found 221.1897.

To a solution of **16** (227 mg, 1.04 mmol) in dry benzene (10 mL) under argon atmosphere, was added 14 mg of VO(acac)_2_. The reaction mixture was stirred for 10 min at refluxing temperature. Then, 0.37 mL of t-BuOOH (3.2 mmol) was added. The reaction was stirred for 30 min at reflux temperature, and monitored by thin-layer chromatography. Upon consumption of the starting material, the mixture was diluted with 180 mL of EtOAc, washed with a saturated solution of NaHCO_3_ (3 x 50 mL), brine (3 x 50 mL), dried over Na_2_SO_4_ and evaporated in vacuo. The crude product was purified by flash chromatography (H:MTBE, 3:2) to obtain 100 mg of 7,11-9,10-diepoxigermacr-4,5-en-8-ol (**17**) (39%). ^1^H-NMR (500 MHz, CDCl_3_): δ 4.97 (d, *J* = 12.4 Hz, 1H), 3.56 (d, *J* = 9.2 Hz, 1H), 2.80 (dd, *J* = 9.2, 0.9 Hz, 1H), 2.61 (dd, *J* = 13.9, 12.5 Hz, 1H), 2.50 (s, 1H), 2.26–2.12 (m, 3H), 1.91–1.74 (m, 3H), 1.67 (s, 3H), 1.65 (s, 3H), 1.48 (s, 3H), 1.37 (s, 3H), 1.32–1.26 (m, 1H). ^13^C-NMR (125 MHz, CDCl_3_): δ 138.22, 119.77, 69.42, 67.03, 66.89, 63.28, 62.48, 36.81, 31.86, 27.91, 22.29, 22.03, 21.95, 21.44, 15.29. HRMS (ESI+) *m*/*z* calculated to C_15_H_25_O_3_ [M + H]^+^ 253.1804, found 253.1794.

#### 3.2.5. Cyclization of germacrone (**1**) with GaCl_3_

To a solution of **1** (150 mg, 0.69 mmol) in dry DCM (7 mL) under argon atmosphere, 12 mg of GaCl_3_ (0.069 mmol) were added. The reaction mixture was stirred for 40 min at room temperature and monitored by thing layer chromatography. Upon consumption of the starting material the reaction was evaporated in vacuo. The crude product was purified by flash chromatography (H:MTBE, 95:5) to obtain 22 mg of eudesma-4,7(11)-dien-8-one (**20**) (15%), 37 mg of eudesma-3,7(11)-dien-8-one (**21**) (24%) and 4 mg of eudesma-4(15),7(11)-dien-8-one (**22**) (3%). Compounds **20** and **21** were previously described by some of us [7].

#### 3.2.6. Epoxidation of eudesma-4,7(11)-dien-8-one (**20**) with NaOH and H_2_O_2_

To a solution of **20** (298 mg, 1.37 mmol) in MeOH (45 mL), was added 1 mL of 2N NaOH and 2.7 mL of H_2_O_2_ (30% in water) at 0 °C. The reaction mixture was stirred at this temperature for 22 h and monitored by thin-layer chromatography. Upon consuming of the starting material, the mixture was diluted with 155 mL of MTBE, washed with brine (3 × 50 mL), dried over Na_2_SO_4_ and evaporated in vacuo. The crude product was purified by flash chromatography (H:MTBE, 4:1) to obtain 137 mg of 7,11-epoxyeudesma-4,7(11)-dien-8-one (**23**) (36%). ^1^H-NMR (500 MHz, CDCl_3_): δ 2.77 (d, *J* = 17.3 Hz, 1H), 2.68 (d, *J* = 17.3 Hz, 1H), 2.37 (s, 2H), 2.04–1.95 (m, 2H), 1.79–1.61 (m, 2H), 1.59 (s, 3H), 1.56–1.48 (m, 1H), 1.43 (s, 3H), 1.42–1.36 (m, 1H), 1.31 (s, 3H), 1.14 (s, 3H). ^13^C-NMR (125 MHz, CDCl_3_): δ 208.00, 128.77, 128.52, 68.32, 65.64, 53.78, 37.83, 35.01, 31.65, 29.81, 26.54, 20.12, 19.32, 19.17, 18.63. HRMS (ESI+) *m*/*z* calculated to C_15_H_23_O_2_ [M + H]^+^ 235.1698, found 235.1687.

#### 3.2.7. Epoxidation of eudesma-3,7(11)-dien-8-one (**21**) with NaOH and H_2_O_2_

To a solution of **21** (98 mg, 0.45 mmol) in MeOH (15 mL), was added 0.5 mL of 2N NaOH solution and 0.8 mL of H_2_O_2_ (30% in water) at a temperature of 0 °C. The reaction mixture was stirred at that temperature for 22 h and monitored by thin-layer chromatography analysis. Upon consumption of the starting material, the mixture was then diluted in 60 mL of MTBE, washed with brine (3 × 20 mL), dried over Na_2_SO_4_ and evaporated in vacuo. The crude product was purified by flash chromatography (H:MTBE, 4:1) to obtain 42 mg of 7,11-epoxyeudesma-3,7(11)-dien-8-one (**24**)(40%). ^1^H-NMR (500 MHz, CDCl_3_): δ 5.43 (bs, 1H) 2.40–2.31 (m, 2H), 2.20 (d, *J* = 17.7 Hz, 1H), 2.11 (d, *J* = 6.0 Hz, 1H), 2.09–2.01 (m, 2H), 1.96–1.90 (m, 1H), 1.63 (s, 3H), 1.53–1.50 (m, 1H), 1.46–1.42 (m, 1H), 1.41 (s, 3H), 1.27 (s, 3H), 0.99 (s, 3H). ^13^C-NMR (126 MHz, CDCl_3_): δ 208.20, 133.15, 122.26, 68.15, 65.19, 54.62, 43.23, 37.01, 33.63, 29.48, 22.46, 21.13, 20.68, 19.61, 18.62. HRMS (ESI+) *m*/*z* calculated para C_15_H_23_O_2_ [M + H]^+^ 235.1698, found 235.1697.

### 3.3. Computational Analysis

A conformational search with Molecular Mechanics (MMFF94) was carried out with the Macromodel Package [25]. Geometry optimizations and energy calculations were performed with Gaussian 16 [26] using DFT [38,39,40] at the B3LYP/6-31+G(d,p) [27,28] level of theory. Frequency calculations (at 298.15 K) at the same level of theory were used to confirm the nature of all stationary points. Transition states were identified by the presence of a single imaginary frequency. To verify that the TSs correspond to the expected interchange between conformers, intrinsic reaction coordinate (IRC) [41,42,43] calculations were performed at the same level, B3LYP/6-31+G(d,p). The reported energies are expressed in kcal/mol and correspond to relative free energies, while those that appear in the IRC plot, expressed also in kcal/mol, correspond to relative electronic energies, and do not include zero-point energy corrections.

For computation of ^1^H–^1^H coupling constants, we have applied the scaled proton-proton recommendations by Bally T and Rablen [(B3LYP/6-31G(d,p)//B3LYP/b3lyp/6-31g(d)], through a multiplication of the calculated Fermi contact terms by 0.9155 [29].

### 3.4. Biological Evaluation

*S. littoralis, M. persicae* and *R. padi* colonies were reared on artificial diet [44], bell pepper (*Capsicum annuum*) and barley (*Hordeum vulgare*) plants, respectively. The plants are grown from seeds in pots with commercial substrate. The plants for rearing aphids are infected regularly (bell pepper plants with 4 leaves, barley plants of 10 cm length). The insect colonies and host plants were maintained at 22 ± 1 °C, >70% relative humidity with a photoperiod of 16:8 h (L:D) in a growth chamber.

#### 3.4.1. Antifeedant Activity

The upper surface of *C. anuum* and *H. vulgare* leaf disks or fragments (1.0 cm^2^) were treated with 10 μL of the test substance. The crude extracts and products were tested at an initial dose of 100 or 50 µg/cm^2^, respectively. Five Petri dishes (9 cm diameter) or twenty ventilated plastic boxes (2 × 2 cm) with two newly molted *S. littoral* is L6 larvae (<24 h) or ten apterous aphid adults (24–48 h old), each were allowed to feed at room temperature for *S. littoralis* (<2 h), or in a growth chamber for the aphids (24 h, environmental conditions as above). Each experiment was repeated 2–3 times (SE < 10%) and terminated when the consumption of the control disks reached 65–75% for *S. littoralis*, or after 24 h for the aphids. The leaf disk area consumed was measured on their digitalized images (Image J, http://imagej.nih.gov/ij). Settling was measured by counting the number of aphids settled on each leaf fragment. Feeding or settling inhibition (%FI or %SI) was calculated as % FI/%SI = [1 − (T/C) × 100], where T and C are the consumption/settling of the treated and control leaf disks, respectively. The antifeedant effects (% FI/SI) were analyzed for significance by the nonparametric Wilcoxon signed-rank test.

Extracts and compounds with an FI/SI > 75% were further tested in a dose-response experiment (3–4 serial dilutions) to calculate their relative potency (EC_50_, the effective dose to give a 50% feeding/settling reduction) from linear regression analysis (% FI/SI on Log-dose) [45].

#### 3.4.2. Ixodicidal Activity

*H. lusitanicum*-engorged female ticks were collected in central Spain (Finca La Garganta, Ciudad Real) from their host (deer) and maintained at 22–24 °C and 70% RH until oviposition and egg hatch. Resulting larvae (4–6 weeks old) were used for the bioassays [35]. Briefly, 50 µL of test solution were added to 25 mg of powdered cellulose at different concentrations, and the solvent was evaporated. For each test, three replicates with 20 larvae each were used. Dead ticks were counted after 24 h of contact with the treated cellulose at the environmental conditions described, using a binocular magnifying glass. The larvicidal activity data are presented as percent mortality corrected according to Schneider-Orelli’s formula. Effective lethal doses (LC_50_ and LC_90_) were calculated by Probit Analysis (5 serial dilutions, STATGRAPHICS Centurion XVI, version 16.1.02).

## 4. Conclusions

Starting from the natural product germacrone (**1**), available from cultivated *G. macrorrhizum*, we have synthesized up to 23 derivatives in 1–3 steps by achieving different cyclizations and by varying the oxidation state of germacrone (**1**). Some of the synthesized substances are natural products. With these derivatives, we performed ixodicidal tests against *H. lusitanicum,* and antifeedant tests against *S. littoralis*, *M. persicae*, and *R. padi*. The most relevant results were obtained with the epoxyeudesmene **24**, with four times higher ixodicidal activity than germacrone (**1**). Additionally, 1,10-epoxygermacrone (**2**) is one thousand times a more potent antifeedant than germacrone (**1**) against *R. padi*, therefore indicating that this molecule is a promising selective antifeedant against this cereal pest.

## Figures and Tables

**Table 1:** Comparison between the theoretical coupling constant for conformer UU (J_UU_), DD (J_DD_) and UD (J_UD_) and the corresponding experimental values (J_exp_) measured for compound **13**.

	*J*_UU_	*J*_Exp_	*J*_DD_	*J*_Exp_	*J*_UD_	*J*_Exp_
H_1-2a_	3.6	^a^	12.3	12.5	7.6	7.5
H_1-2b_	12.3	12.3	3.9	^a^	9.9	7.5
H_5-6a_	3.6	^a^	10.0	10.7	10.4	10.4
H_5-6b_	12.1	11.9	6.7	7.0	6.3	5.9

^a^ The value of these coupling constants could not be measured accurately, although they must be close to 2 Hz.

**Table 2:** Acaricidal activity of germacrone derivatives against a *H. lusitanicum* Koch.

Compound	% Mortality ^a^	Effective Lethal Doses (mg/mL)	
	10(mg/mL)	LD_50_^b^	LD_90_^b^
**1**	100 ± 0	1.08(0.7–1.38)	3.13(2.66–3.93)
**2**	100 ± 0	2.7(2.43–3.01)	4.1(3.67–4.77)
**3**	98.3 ± 1.7	1.47(1.16–1.76)	3.45(2.48–4.22)
**4**	45.3 ± 24.9	-	-
**5**	8.6 ± 12.1	-	-
**6**	42.6 ± 2.5	-	-
**7**	100 ± 0	2.44(2.08–2.79)	4.47(3.95–5.27)
**8**	100 ± 0	<1.25	<1.25
**9**	-	-	-
**10**	0 ± 0	-	-
**11**	24.6 ± 0.6	-	-
**13**	100 ± 0	1.21(1.04–1.4)	2.43(2.12–2.89)
**14**	100 ± 0	0.96(0.88–1.05)	1.39(1.25–1.56)
**15**	100 ± 0	~2.32	~2.98
**17**	32.1 ± 17.3	-	-
**19**	0 ± 0	-	-
**20**	0	-	-
**21**	0	-	-
**23**	100 ± 0	4.14(3.85–4.48)	6.33(5.82–7.04)
**24**	91.9 ± 0.6	0.23(0.12–0.47)	1.91(1.57–2.54)
**Thymol ^c^**	100	0.18(0.16–0.20)	0.27(0.24–0.31)

^a^ Data corrected according to Schenider Orelli’s formula. ^b^ Doses needed to give 50 and 90% mortality (95% Confidence Limits). ^c^ From Navarro-Rocha et al. [1].

**Table 3:** Antifeedant effects of germacrone derivatives against *S. littoralis*, *M. persicae* and *R. padi*. Effective doses (EC_50_ in mg/mL) have been included for the positive controls azadiractin (AZA) and farnesol (FAR).

Compound	*S. littoralis*		*M. persiscae*		*R. padi*	
	%FI ^a^	EC_50_^b^	%SI ^a^	EC_50_^b^	%SI ^a^	EC_50_^b^
**1**	98.6 ± 0.6	1.9(0.1–3.6)	82.2 ± 5.0	2.8(2.2–3.4)	69.6 ± 6.5	<5
**2**	76.93 ± 6.7	<5	69.0 ± 7.8	<5	97.8 ± 1.0	0.006 (0.27–0.2)
**3**	69.9 ± 11.3	<5	64.0 ± 8.9	<5	94.5 ± 1.7	0.2 (-0.1–0.4)
**6**	-	-	60.6 ± 6.8	<5	-	-
**7**	61.1 ± 11.7	<5	61.6 ± 9.1	<5	97.3 ± 1.3	0.02 (0.006–0.07)
**8**	90.8 ± 3.3	~2.5	75.6 ± 7.2	<5	98.3 ± 1.2	0.8 (0.6–1.0)
**9**	73.8 ± 15.4	<5	91.0 ± 2.6	<5	-	-
**10**	78.6 ± 12.7	<5	88.5 ± 3.1	<5	-	-
**11**	54.3 ± 8.71	-	45.0 ± 9.1	-	38.44 ± 8.8	-
**13**	54.6 ± 9.4	-	66.1 ± 7.4	<5	92.7 ± 2.2	2.3 (2.1–2.5)
**14**	49.8 ± 11.3	-	71.4 ± 6.1	<5	84.4 ± 6.5	<5
**15**	54.0 ± 11.0	-	75.0 ± 5.1	1.0(0.01–1.4)	69.3 ± 7.2	3.69 (3.3–4.0)
**17**	79.7 ± 7.1	<5	50.4 ± 9.3	-	86.0 ± 4.5	1.8 (1.4–2.1)
**23**	53.9 ± 9.6	-	55.8 ± 9.8	-	87.0 ± 4.5	1.5 (1.1–1.9)
**24**	33.9 ± 15.6	-	48.5 ± 9.2	-	52.5 ± 8.8	-
**AZA ^c^ RY**	0.5 × 10^−6^ (0.2 × 10^−8^–0.7 × 10^−4^)		-		-	
**FAR ^d^**	-		14.9 (12.1–18.3)		1.7 (1.2–2.7)	

^a^ %FI/SI = (1−(T/C)) × 100, where T and C are feeding/settling on treated and control leaf disks at a dose of 5 mg/mL. ^b^ EC_50_, effective dose (mg/mL) to give a 50% inhibition (95% Confidence Limits). ^c^ From González-Coloma et al. [36]. ^d^ From Santana et al. [37].

## Supplementary Materials

The supplementary material related to this article includes NMR spectra of new compounds is available online at https://www.mdpi.com/1420-3049/24/16/2898/s1.
