# Supplementary material for: Germacrone Derivatives as new Insecticidal and Acaricidal Compounds: A Structure-Activity Relationship
Source: Molecules. 2019 Aug 9;24(16):2898. doi: 10.3390/molecules24162898 (PMC6720995; doi:10.3390/molecules24162898)

Germacrone derivatives as new insecticidal and acaricidal compounds: Structure-Activity Relationship

Alberto Galisteo Pretel[1], Helena Pérez del Pulgar[1], Estela Guerrero de León[2], José Luis López-Pérez[2,3], A. Sonia Olmeda[4], Azucena Gonzalez-Coloma[5], Alejandro F. Barrero[1,*] and José Francisco Quílez del Moral[1,*]

[1] Department of Organic Chemistry, Institute of Biotechnology, University of Granada, 18071 Granada, Spain; albertogapre@ugr.es (A.G.P.); helenaperezpv@ugr.es (H.P.P.)

[2] Department of Pharmacology, Faculty of Medicine, University of Panama, Republic of Panama; guerrerodleon@gmail.com; (E.G.L.)

[3] Department of Pharmaceutical Sciences, IBSAL-CIETUS, University of Salamanca, 37007 Salamanca, Spain; lopez@usal.es (J.L.L.P.)

[4] Faculty of Veterinary, Complutense University of Madrid (UCM), 28040 Madrid, Spain; angeles@ucm.es (A.S.O.)

[5] Institute of Agricultural Sciences, CSIC, 28006, Madrid, Spain; azu@ica.csic.es (A.G.C.)

* Correspondence: afbarre@ugr.es; jfquilez@ugr.es; Tel.: +34-958243185

List of contents

NMR spectra 2-8

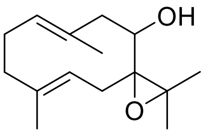

12

^{1}H NMR (400 MHz, $CDCl_3$)

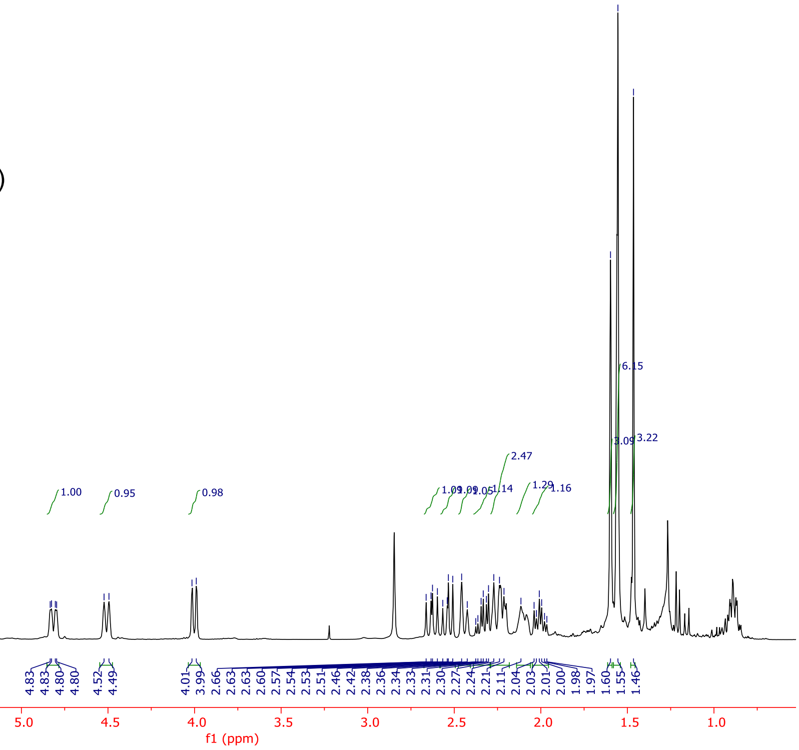

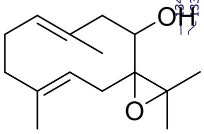

12

^{13}C NMR (100 MHz, $CDCl_3$)

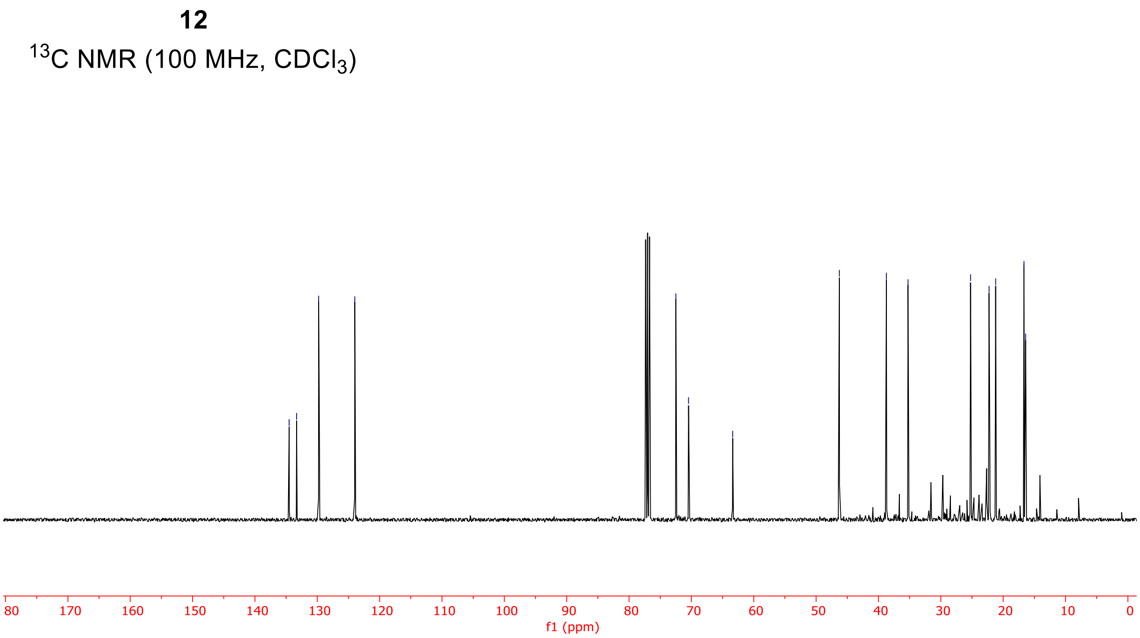

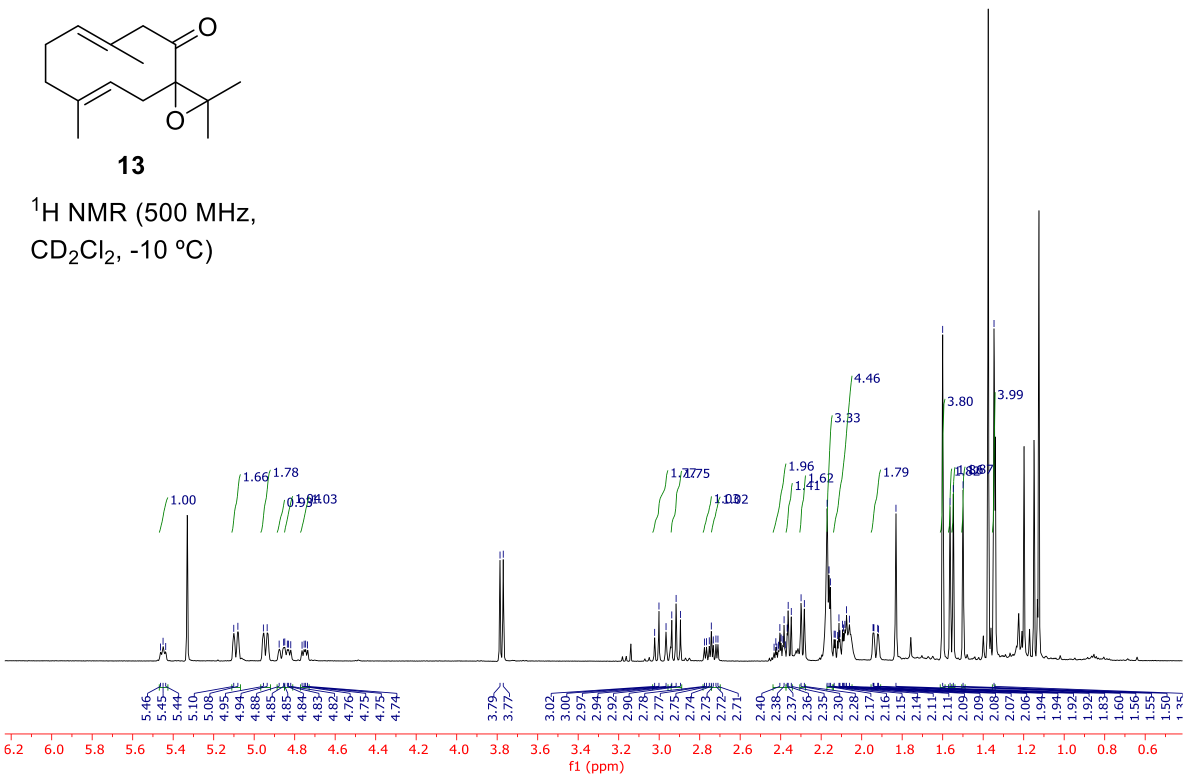
13
^{1}H NMR (500 MHz, CD_2Cl_2, -10 ºC)
f1 (ppm)

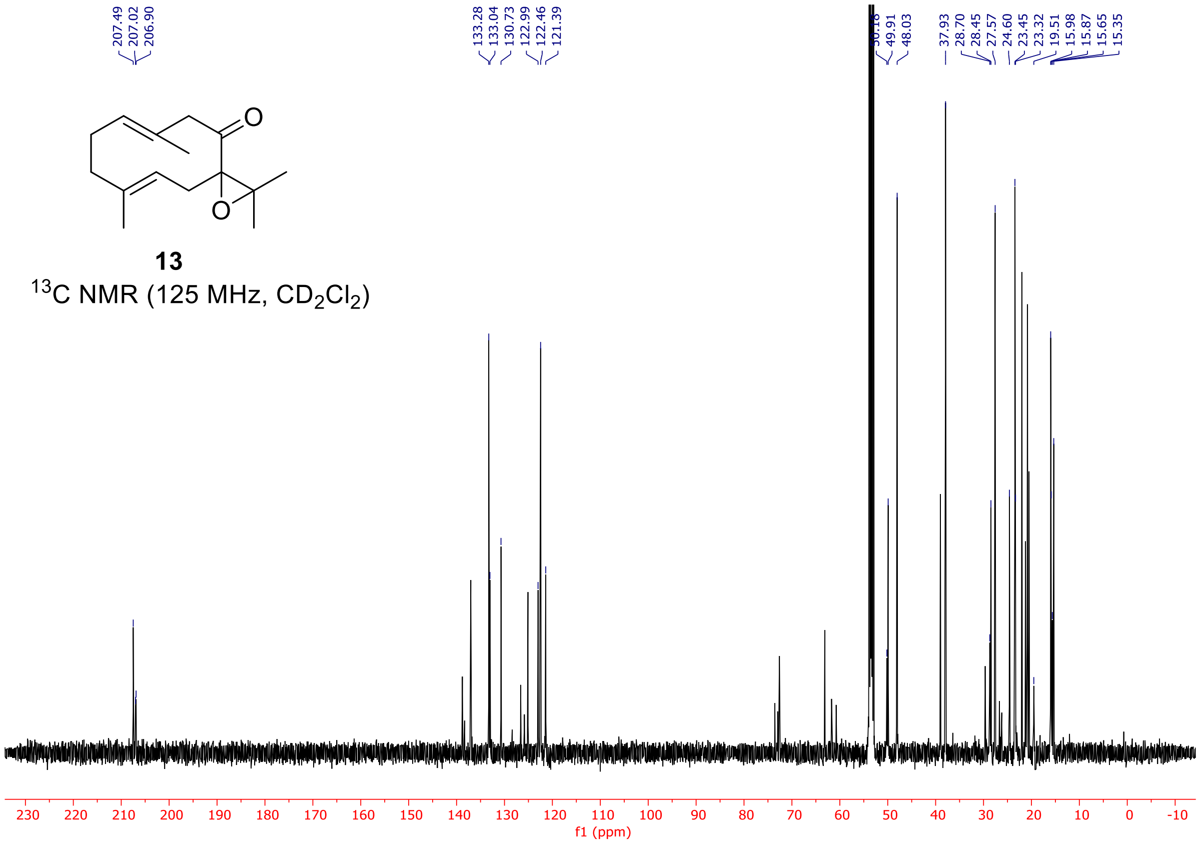
13
^{13}C NMR (125 MHz, CD_2Cl_2)
f1 (ppm)

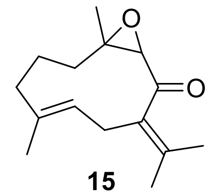

15

^{1}H NMR (500 MHz, DMSO-d_6, 85 ºC)

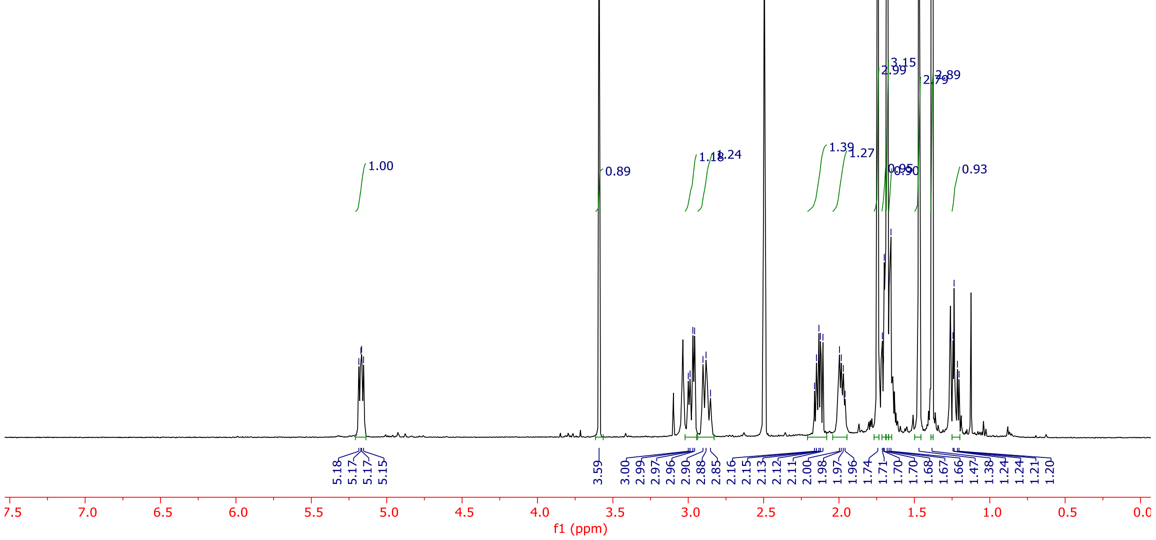

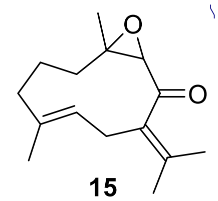

15

^{13}C NMR (125 MHz, DMSO-d_6, 85 ºC)

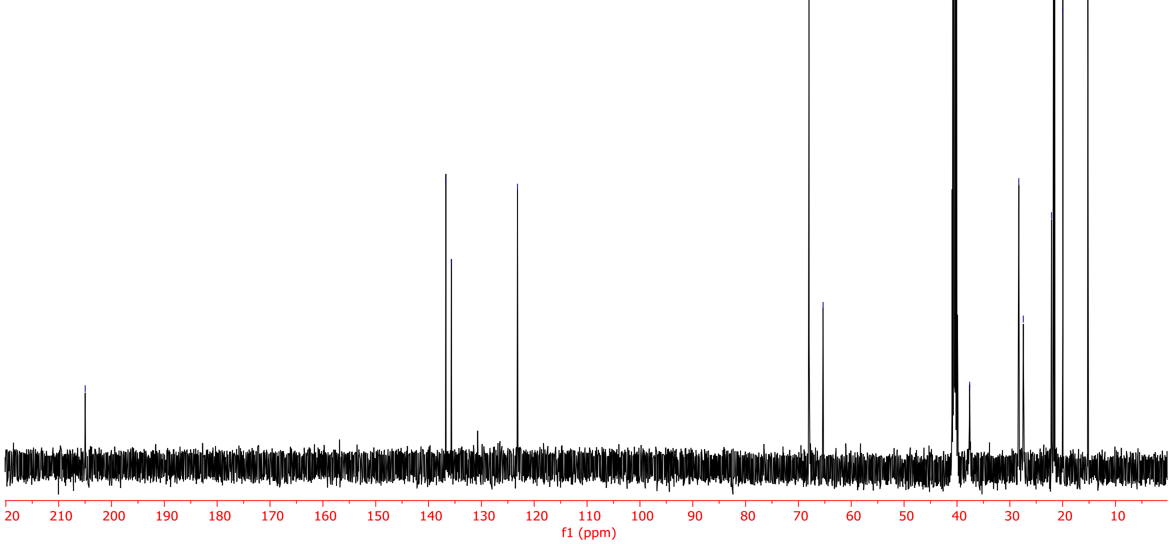

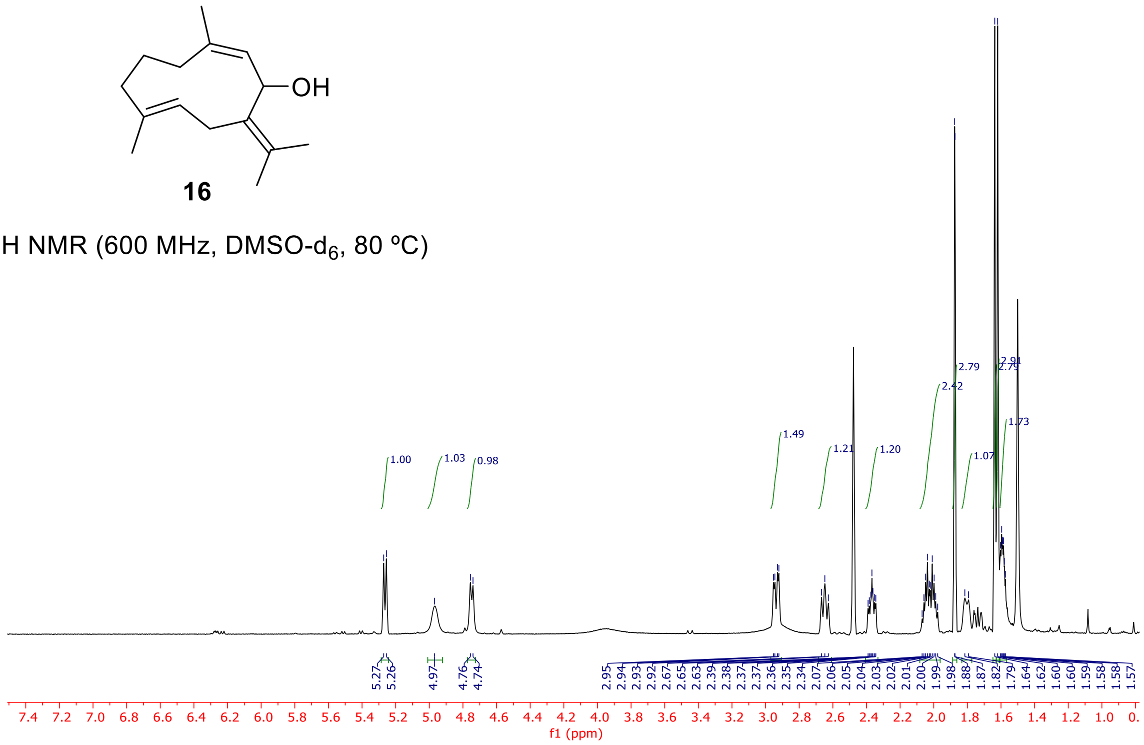

16

^{1}H NMR (600 MHz, DMSO-d_6, 80 ºC)

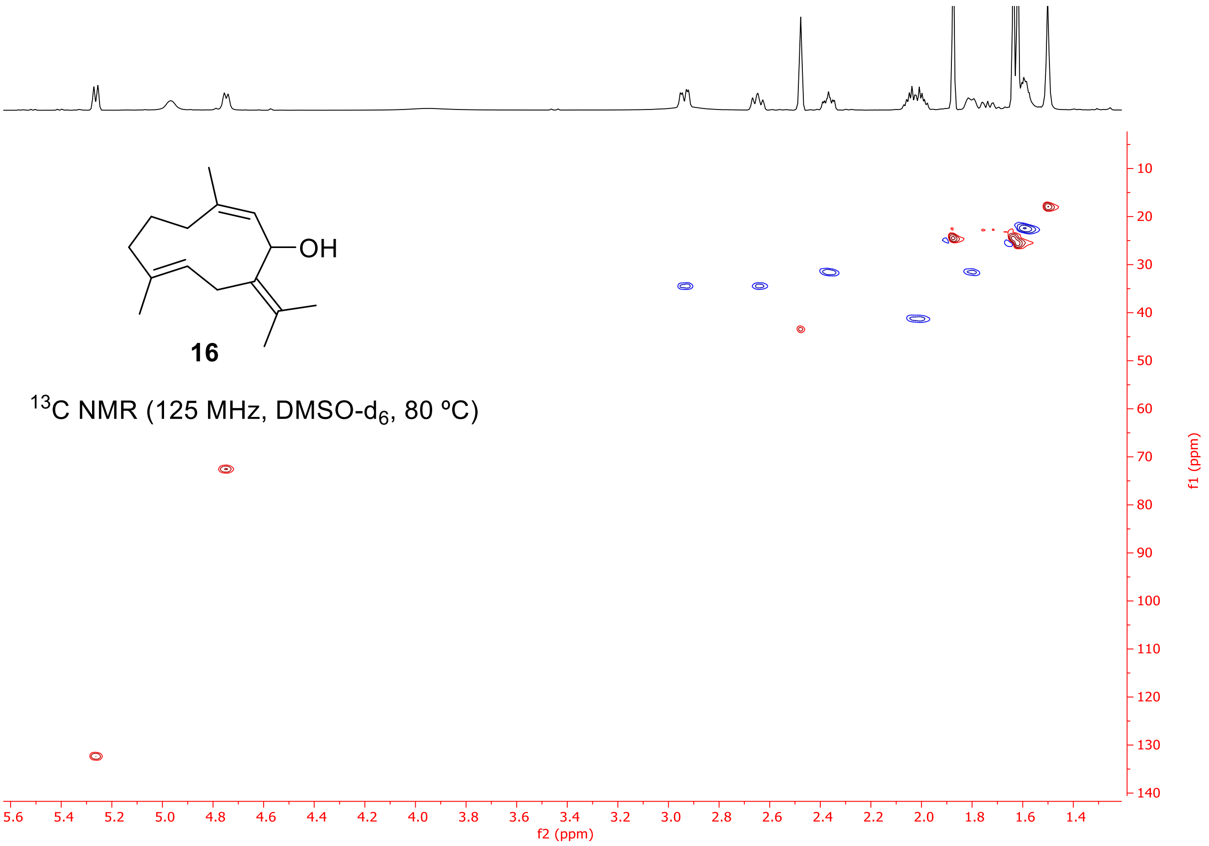

16

^{13}C NMR (125 MHz, DMSO-d_6, 80 ºC)

17

^{1}H NMR (500 MHz, $CDCl_3$)

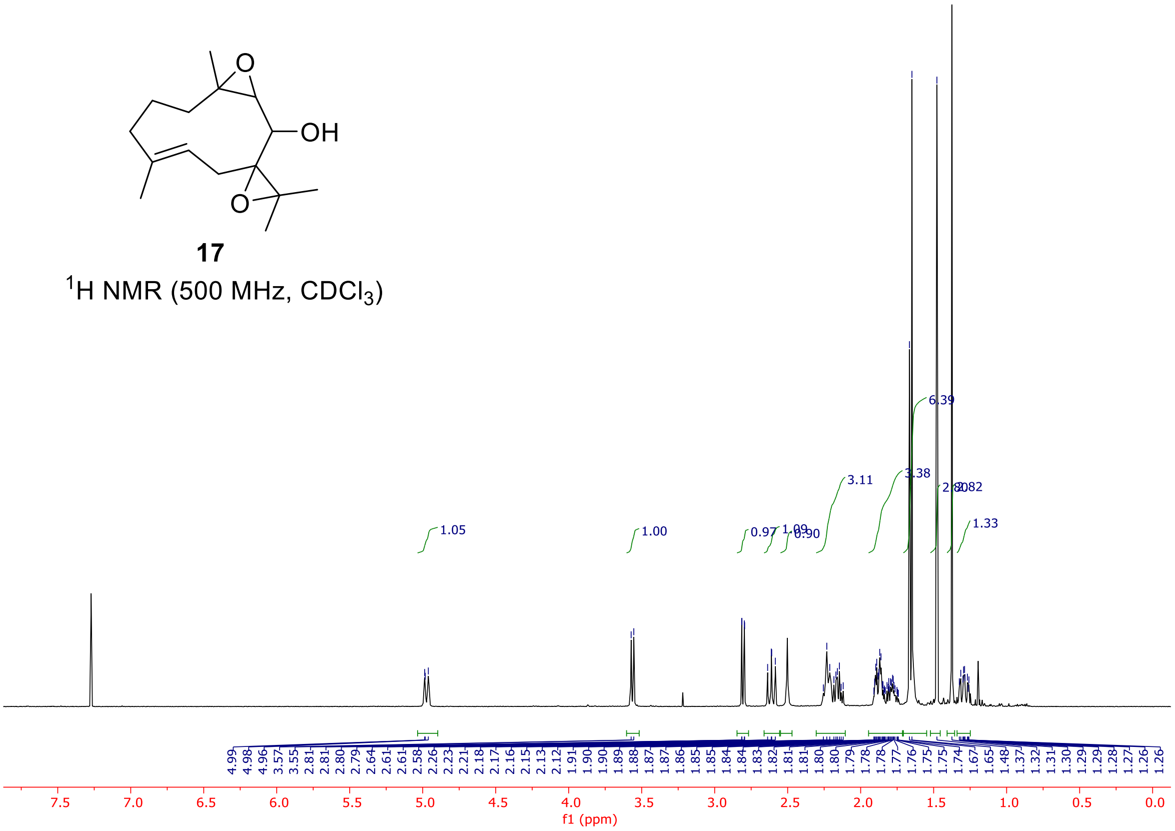

17

^{13}C NMR (125 MHz, $CDCl_3$):

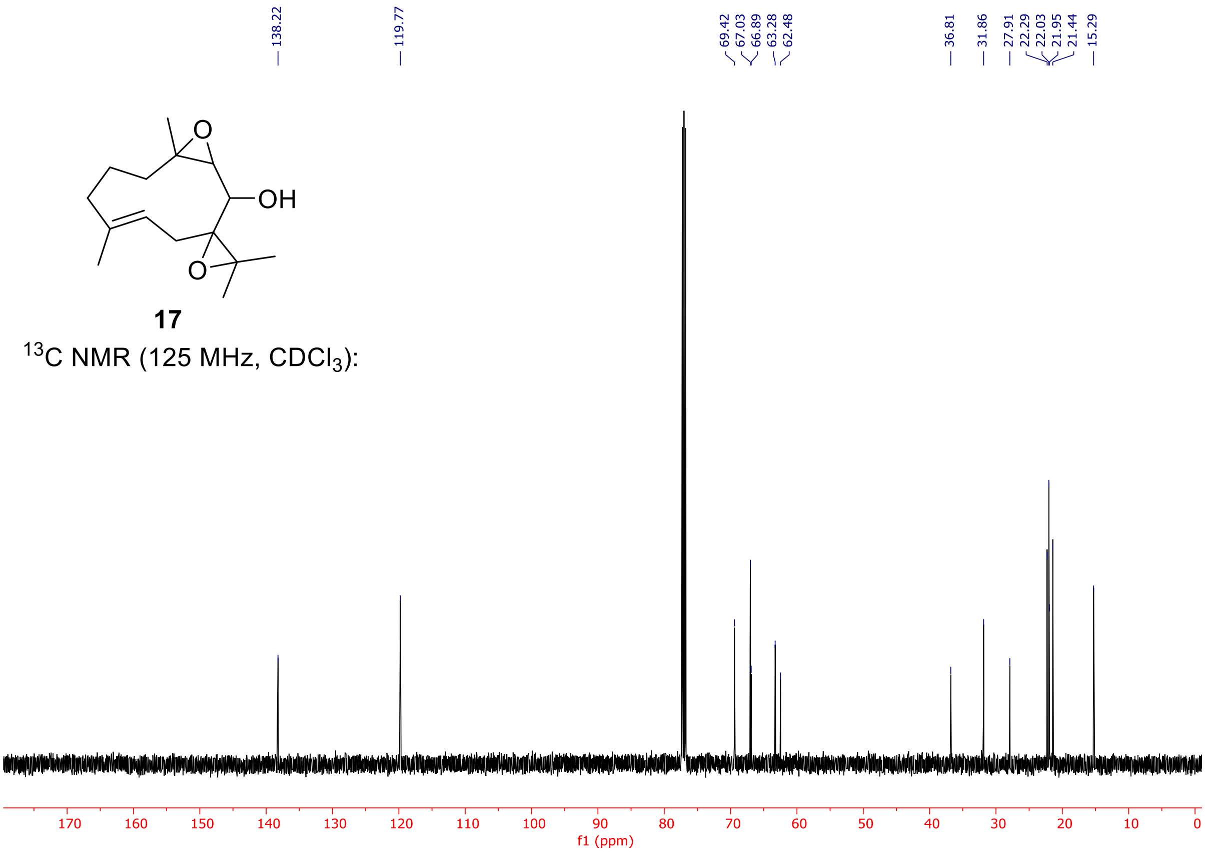

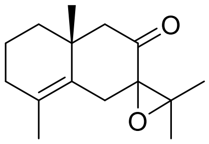

23

^{1}H NMR (500 MHz, $CDCl_3$):

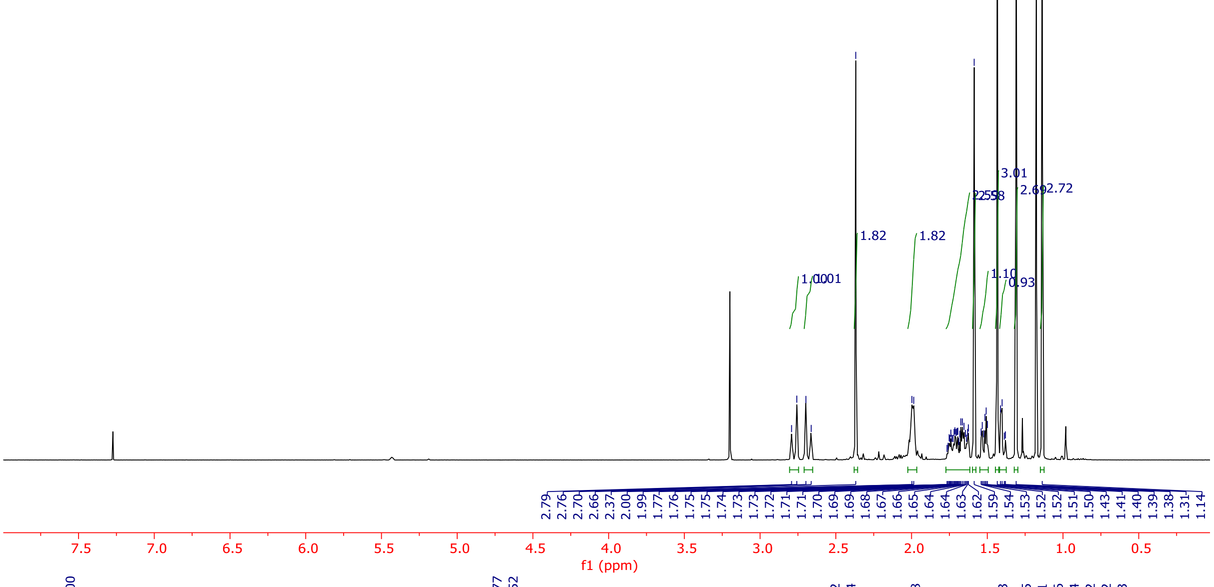

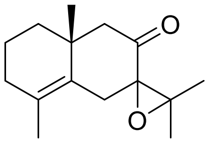

23

^{13}C NMR (125 MHz, $CDCl_3$)

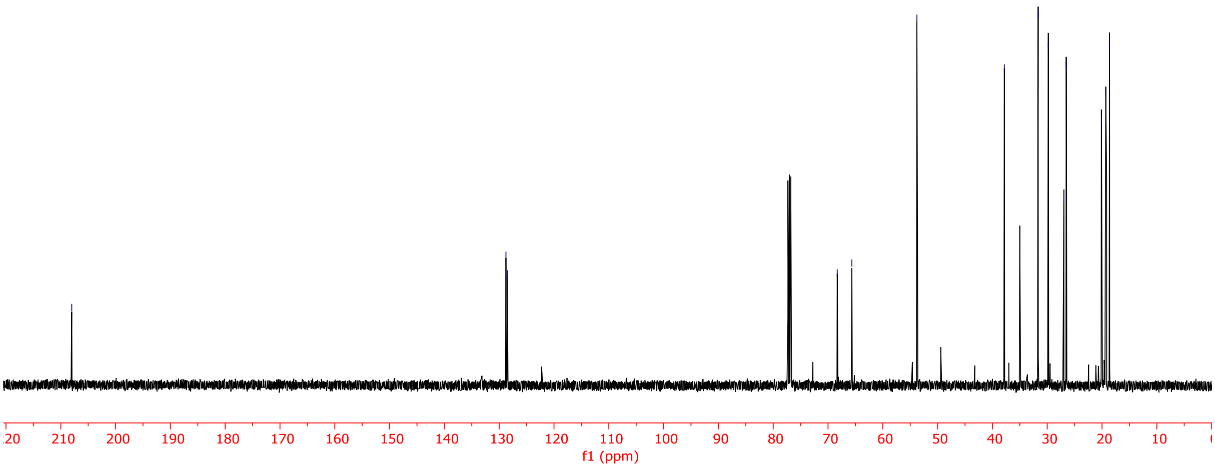

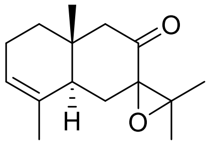

24

^{1}H NMR (500 MHz, $CDCl_3$)

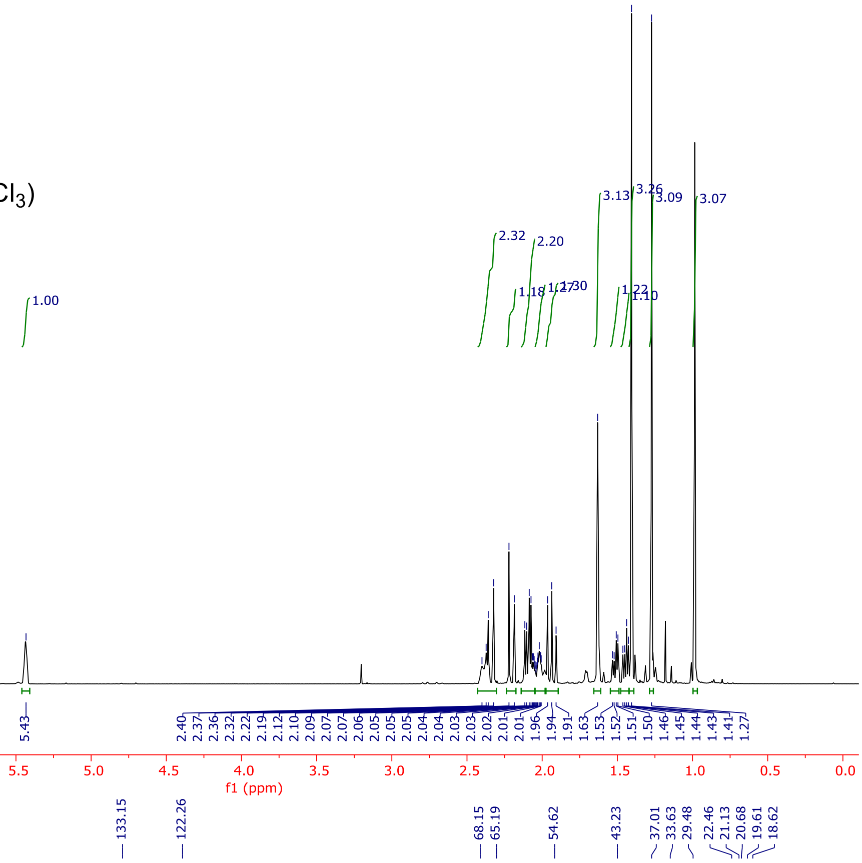

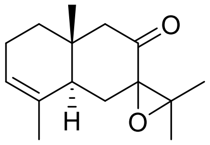

24

^{13}C NMR (126 MHz, $CDCl_3$)

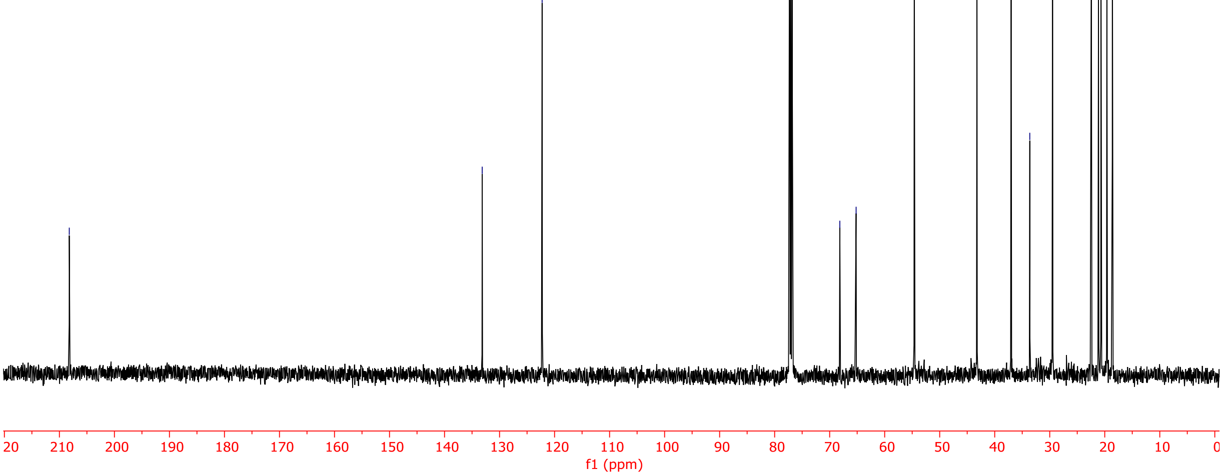

Supplement: Supplementary file 1 [file molecules-24-02898-s001.pdf]
